# Bioassay-guided isolation and in Silico characterization of cytotoxic compounds from *Hemimycale* sp. Sponge targeting A549 lung cancer cells

**DOI:** 10.1186/s13065-024-01325-w

**Published:** 2024-11-01

**Authors:** Asmaa Abo Elgoud Said, Islam M. Abdel-Rahman, Yaser A. Mostafa, Eman Zekry Attia, Mamdouh Nabil Samy, Usama Ramadan Abdelmohsen, Katsuyoshi Matsunami, Mostafa A. Fouad, Yaser G. Gouda

**Affiliations:** 1 Department of Pharmacognosy, Faculty of Pharmacy, Minia University, Minia, 61519 Egypt; 2 Department of Pharmaceutical Chemistry, Faculty of Pharmacy, Deraya University, New-Minia, 61111 Egypt; 3 Pharmaceutical Organic Chemistry Department, Faculty of Pharmacy, Assiut University, Assiut, 71526 Egypt; 4 Department of Pharmacognosy, Faculty of Pharmacy, Deraya University, Universities Zone, New Minia City, 61111 Egypt; 5 Graduate School of Biomedical and Health Sciences, Hiroshima University, 1–2–3 Kasumi, Minami-ku, Hiroshima, 734–8553 Japan; 6 Department of Pharmacognosy, Faculty of Pharmacy, Assiut University, Assiut, 71526 Egypt

**Keywords:** Red sea, Marine sponge, *Hemimycale*, Cytotoxicity, NMR, In silico

## Abstract

Bioassay-guided fractionation approach led to identification of two novel compounds; (4-(hydroxymethyl)-3-methoxy-1*H*-pyrazol (**1**) and mycalene (**2**), alongside with four known metabolites; octadecane (**3**), hexatriacontane (**4**), 1-heneicosanol (**5**) and heptatriacontanoic acid (**6**) from the Red Sea marine sponge *Hemimycale* sp. The ethyl acetate fraction showed a noticeable cytotoxic activity against the lung cancer cell line (A549) with IC_50_ value of 75.54 µg/ mL. Structural elucidation was achieved using a combination of 1D and 2D nuclear magnetic resonance (NMR) spectroscopy and high-resolution electrospray ionization-mass spectrometry (HR-ESI-MS). To elucidate the potential mechanism of action behind the cytotoxic effects against lung cancer, a multi-faceted approach combining in silico network pharmacology, experimental validation, and molecular docking studies were employed. Both compounds, designated as 1 and 2, demonstrated significant binding affinities to predicted target proteins, with docking scores of -4.789 and − 4.421 kcal/mol, respectively. These results lay the groundwork for further investigation into the therapeutic potential of these novel compounds from *Hemimycale* sp. as promising candidates for lung cancer treatment.

**Supplementary Information:**

The online version contains supplementary material available at 10.1186/s13065-024-01325-w.

## Introduction

Nowadays, the development of new medicines is an essential challenge. The marine habitat is an attractive field for isolation and identification of natural pharmaceuticals due to its relatively unknown richness compared to terrestrial ecosystems [1, 2]. Organisms that inhabit the aquatic environment provide an extensive biological and chemical variety as these circumstances are totally different from terrestrial environment whether in terms of temperature, pressure, light, or nutritional factors. The unique living conditions make the marine habitat particularly fascinating for the development of novel compounds since these conditions necessitate distinct adaption mechanisms such as the generation of physiologically active secondary metabolites [3, 4]. Furthermore, the development of new chemical and physical techniques has led to isolation and structural elucidation of new minor marine secondary metabolites [5]. For decades, pharmaceutical corporations and academic institutes have invested heavily in isolating and discovering novel marine metabolites. Bergmann led the first exploratory trips to find marine biologically active metabolites in the 1950s and identified the first two bioactive nucleosides, spongouridine and spongothymidine, from the sponge *Cryptotethia crypta* [6, 7]. These nucleosides served as the starting point for the synthesis of Ara-A and Ara-C (or Cytarabine), which has been the primary therapy for acute myelogenous leukaemia for over thirty years [6, 8, 9].

Currently, cancer is one of the most deadly illnesses in the world. World Health Organization predicts 21 million additional cancer diagnoses and 13 million deaths by 2030 [10]. The most common malignancies include colon, breast, prostate, lung, rectal, skin, and stomach. Lung cancer accounted for the majority of cancer-related fatalities in 2020 [11].

The marine sponges belonging to family Hymedesmiidae afforded different metabolites which exhibited cytotoxic activity against various cell lines [12]. The marine sponge *Hemimycale* sp. was afforded several bioactive metabolites, including alkaloids with distinct structures which displayed significant cytotoxic activity against various cell lines [13, 14]. Consequently, we investigated the cytotoxic activity of different fractions of the organic extract of the Red Sea marine sponge *Hemimycale* sp., against a lung cancer cell line, isolated and identified the active metabolites as part of our ongoing search for bioactive compounds from this marine sponge. This investigation led to isolation and identification of two new compounds, along with four previously reported ones. Subsequently, a comprehensive approach, incorporating* in silico* network pharmacology predictions, experimental validation, and molecular docking analyses, was utilized to investigate the potential mechanism of action.

## Results and discussion

### Cytotoxic assay

In previous work, the total methanolic extract of the marine sponge was tested on several cancer cell lines as well as a normal lung cell line (Wi38) and exhibited IC_50_ value of 367.43 µg/ mL [15]. Moreover, in this study it was tested against lung cancer cell line (A549) and revealed noticeable activity. Different fractions were subsequently tested against lung cancer cell line, where the ethyl acetate fraction showed the highest activity, with an IC_50_ value of 75.54 µg/ mL (Table S1).

### Identification of the isolated compounds

**Fig. 1:**
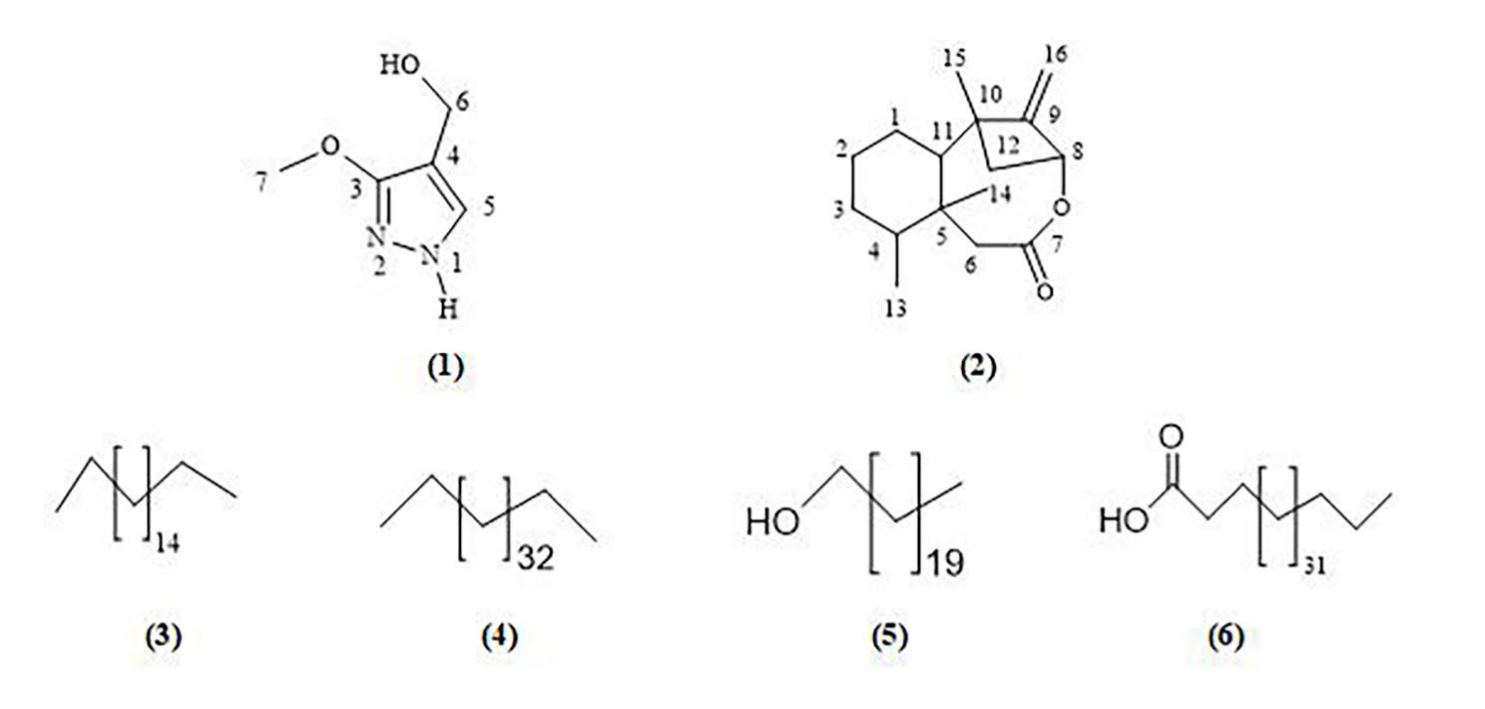
Isolated compounds from marine sponge *Hemimycale* sp

Two new compounds, 4-(hydroxymethyl)-3-methoxy-1*H*-pyrazol (**1**) and mycalene (**2**) were isolated from the ethyl acetate fraction using a bioassay-guided isolation strategy; besides, four known compounds, octadecane (**3**), hexatriacontane (**4**), 1-heneicosanol (**5**) and heptatriacontanoic acid (**6**), which were isolated from the 50% petroleum ether in ethyl acetate (Fig. 1). The structures of the isolated compounds were identified by spectroscopic investigations including NMR data and mass spectrometry.

#### Compound 1

The negative HR-ESI-MS of compound **1** showed a *pseudo* molecular ion peak at *m/z* 127.0405 [M-H]^−^ in consistence with the molecular formula C_5_H_7_N_2_O_2_ (Fig. S3). The ^1^H and ^13^C NMR spectroscopic data (Figs. S1-2) and (Table 1) indicated that compound **1** was substantially identical to a previously isolated pyrazole derivative [16] except for the presence of a methoxy moiety at *δ*_C_ 52.5 (C-7) / *δ*_H_ 3.87 (3 H, s, H_3_-7). The chemical shift of the methoxy group as well as the chemical shift of other carbon signals verified that the hydroxyl group at C-3 of previously reported pyrazole compound is substituted by the methoxy group [17]. The structure was determined to be 4-(hydroxymethyl)-3-methoxy-1*H*-pyrazol which is a new pyrazole derivative.

**Table 1:** ^1^H and ^13^C NMR spectroscopic data of compound **1** (500 and 125 MHz, CDCl_3_, respectively)

No.	δ_H,_ integr, mult.	δ_C_, type
3	-	165.3 s
4	-	nd*
5	8.04, 1 H, s	129.8 d
6	4.62, 2 H, s	62.8 t
7	3.87, 3 H, s	52.5 q

nd* Not detected

**Fig. 2:**
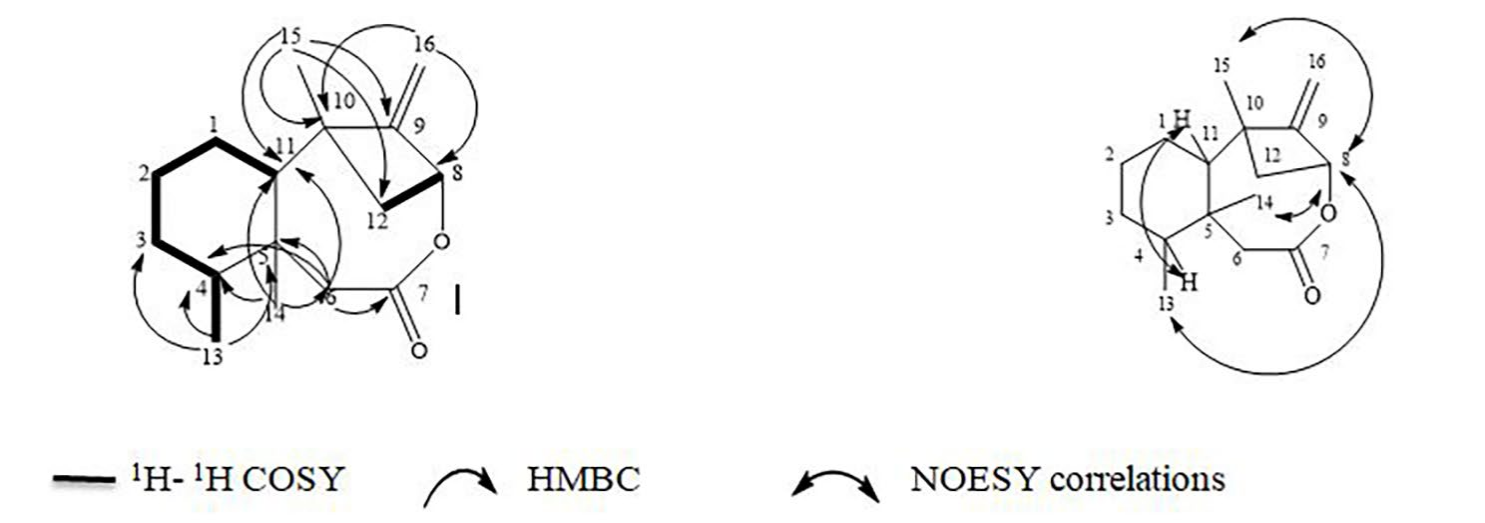
Key ^1^H-^1^H COSY, HMBC, and NOESY correlations of compound **2**

**Compound 2**: [α]_D_^25^ − 185 (*c* = 0.2, CHCl_3_). The FTIR spectrum (Fig. S14). exhibited two prominent absorption bands corresponding to C-H stretching and ester linkage stretching at 2927 and 1712 cm^-1^, respectively. Moreover, the molecular formula was established as C_16_H_24_O_2_ from its HR-ESI-MS spectrum as it showed a *pseudo* molecular ion peak at *m/z* 249.1847 [M + H]^+^ indicating five degrees of unsaturation (Fig. S13). The ^13^C NMR and DEPT experiment showed sixteen signals equivalent to sixteen carbon atoms including 3 methyls, 6 methylenes, 3 methines and 4 quaternary carbon atoms. The ^1^H and ^13^C NMR, DEPT and HSQC data (Figs. S4-8 and S10). confirmed these groups as follow: signals at *δ*_H_ 0.86 (s, 3 H) /*δ*_C_ 17.2; at *δ*_H_ 1.06 (s, 3 H)/*δ*_C_ 21.0, and at *δ*_H_ 0.94 (d, J = 6.6 Hz)/*δ*_C_ 16.2 for the three methyl groups at C-14, 15 and 13, respectively. The methine groups were deduced from the signals at *δ*_H_ 4.32 (m)/*δ*_C_ 69.7, at *δ*_H_ 1.34 (m)/ *δ*_C_ 49.5 and at *δ*_H_ 1.79 (m)/*δ*_C_ 37.6 for atoms at C-8, 11 and 4, respectively. The remaining methylene groups were deduced from the signals at *δ*_H_ 4.75 and 4.94 (each 1H. br s)/*δ*_C_ 99.7; at *δ*_H_ 1.64 (2 H, m)/*δ*_C_ 37.0, at *δ*_H_ 1.92 (m)/*δ*_C_ 21.2, at *δ*_*H*_ 1.52 (2 H, each m)/ *δ*_C_ 27.1, at *δ*_*H*_ 2.36 and 2.40 ( each 1H, d, J = 13.6 Hz)/*δ*_C_ 42.4 and at *δ*_*H*_ 1.23 and 2.24 (each 1H, m)/*δ*_C_ 37.3 for atoms at C-16, 1, 2, 2, 3, 6 and 12, respectively. Finally, the quaternary carbons were observed at *δ*_C_ 40.3, 41.2, 161.7 and 175.7 for C-10, 5, 9 and 7, respectively. The interconnectivity of the structure was established from the 2D NMR data obtained from ^1^H to ^1^H COSY and HMBC experiments (Figs. S9 and S11). (Table 2). The ^1^H-^1^H COSY (Fig. 2) showed two spin systems correlations: H-1(*δ*_H_ 1.64)/ H-2(*δ*_H_ 1.92) and H-11 (*δ*_H_ 1.34); H-2 (*δ*_H_ 1.92)/ H-3(*δ*_H_ 1.52); H-3 (*δ*_H_ 1.52)/ H-4(*δ*_H_ 1.79) and finally, H-4 (*δ*_H_ 1.79)/ Me-13(*δ*_H_ 0.94) which is attributed to the spin system of hexagonal ring (A), in addition, the correlation of H-8 (*δ*_H_ 4.32) / H-12a (*δ*_H_ 1.23) and H-12b (*δ*_H_ 2.24) equivalent to spin system of the bicycle bridge between ring B and C. The HMBC (Fig. 2) showed correlations of H-6(a and b) (at *δ*_H_ 2.36 and 2.40, respectively) with C-4 (*δ*_C_ 37.6), C-5 (*δ*_C_ 41.2), C-7 (*δ*_C_ 175.7), and C-11 (*δ*_C_ 49.5) which prove the link between the two fused rings, methylated cyclohexane and heterocyclic octanon ring (oxocanone ring). Moreover, correlations of Me-13 (*δ*_H_ 0.94) with C-3 (*δ*_C_ 27.1), C-4 (*δ*_C_ 37.6) and C-5 (*δ*_C_ 41.2) and of Me-14 (*δ*_H_ 0.86) with C-4 (*δ*_C_ 37.6), C-6 (*δ*_C_ 42.4) and C-11 (*δ*_C_ 49.5) that illustrate position of two methyl groups, Me-13 and Me-14, besides the position of the carbonyl group. While, the correlations from Me-15 (*δ*_H_ 1.06) to C-9 (*δ*_C_ 161.7), C-10 (*δ*_C_ 40.3), C-11 (*δ*_C_ 49.5) and C-12 (*δ*_C_ 37.3) and from H-16(a and b) (*δ*_H_ 4.75 and 4.94, respectively) to C-8 (*δ*_C_ 69.7) and C-10 (*δ*_C_ 40.3) established the position of methyl group, Me-15 at C-10, the position of exomethylene group and also the bridged bicyclic system. The relative stereochemistry was tentatively identified from key correlations in NOESY spectrum (Fig. 2 and S12) as the correlations [H-8↔H-13, H-14 and H-15] indicating that those stereo centers are in the same orientation. On the other hand, the correlation [H-4↔H-11] indicates that they are in the same phase of the compound and are on other side from the other stereo centers due to the molecule rigidity. From the 3D structure and modeling of the compound and according to structure rigidity, the suggested configuration of the five stereo centers are C-4 (*S*), C-5 (*R*), C-8 (*S*), C-10 (*S*) and C-11 (*R*). The above-mentioned data confirmed that compound **2** is a new compound with a unique nucleus and it was namely mycalene.

The 50% petroleum ether in ethyl acetate fraction afforded four known compounds, two hydrocarbons; octadecane (**3)** and hexatriacontane (**4)**, one fatty alcohol; 1-heneicosanol (**5)** and the fatty acid heptatriacontanoic acid (**6).** Their structure was identified from ^1^H, ^13^C and DEPT-135 NMR data, while the length of the aliphatic chains was determined from HR-ESI-MS and EI-MS spectroscopic data (Figs. S15-27).

**Table 2:** NMR spectroscopic data of compound **2** (500 and 125 MHz, CDCl_3_, respectively)

NO.	δ_H,_ integr, mult. (J in H_z_)	δ_C,_ type	HCOSY (^1^H 1 ^1^H)	HMBC (^1^H→^13^C)	NOESY (^1^H 1 ^1^H)
1	1.64, 2 H, m	37.0 t	H_2_, H_11_	-	-
2	1.92, 2 H, m	21.2 t	H_1,_ H_3_	-	-
3	1.52, 2 H, m	27.1 t	H_2,_ H_4_	-	-
4	1.79, 1 H, m	37.6 d	H_3_	-	H_11_
5	-	41.2 s	-	-	
6	6 a: 2.36, 1 H, d (13.6) 6 b: 2.40, 1 H, d (13.6)	42.4 t	-	C-4, C-5, C-7, C-11	-
7	-	175.7 s	-	-	-
8	4.32, 1 H, m	69.7 d	H_12a,_ H_12b_	-	H_13,_ H_14,_ H_15_
9	-	161.7 s	-	-	-
10	-	40.3 s	-	-	-
11	1.34, 1 H, m	49.5 d	H_1_	-	H-4
12	12 a: 1.23, 1 H, m 12 b: 2.24, 1 H, m	37.3 t	H_8_	-	-
13	0.94, 3 H, d (6.6)	16.2 q	H-4	C-3, C-4, C-5	H_8,_ H_14,_ H_15_
14	0.86, 3 H, s	17.2 q	-	C-4, C-6, C-11	H_8,_ H_13,_ H_15_
15	1.06, 3 H, s	21.0 q	-	C-9, C-10, C-11, C-12	H_8,_ H_13,_ H_14_
16	16 a: 4.75, 1 H, brs 16 b: 4.94, 1 H, brs	99.7 t	-	C-8, C-10	-

## Construction of pharmacological network

### Construction of NSCLC-targets network

To help find non-small cell lung cancer (NSCLC)-related genes with our compounds, we proposed a network analysis using bioinformatic tools like DisGeNET, a comprehensive database of disease-associated genes and variants. It facilitates querying, analyzing, and visualizing various network representations of gene-disease and variant-disease associations. Data within DisGeNET originates from diverse sources, encompassing expert-curated archives, repositories of GWAS, animal models, and peer-reviewed scientific publications [18]. The curation of “Adenocarcinoma of lung disorder” resulted in 2438 associated gene, then these genes were analyzed using HumanNet [19], a probabilistic functional gene network utilized in disease research, aiding in the identification of gene-disease-drug associations. Its primary function lies in predicting and organizing genes relevant to specific diseases, each gene–gene association has a score that represents the probability of the association. We employed tissue gene expression data as the gene features sourced from BioGPS [20]. The analysis showed that 142 genes were linked to NSCLC from DisGeNET associated genes [21].

#### Construction of compound target network

**Fig. 3:**
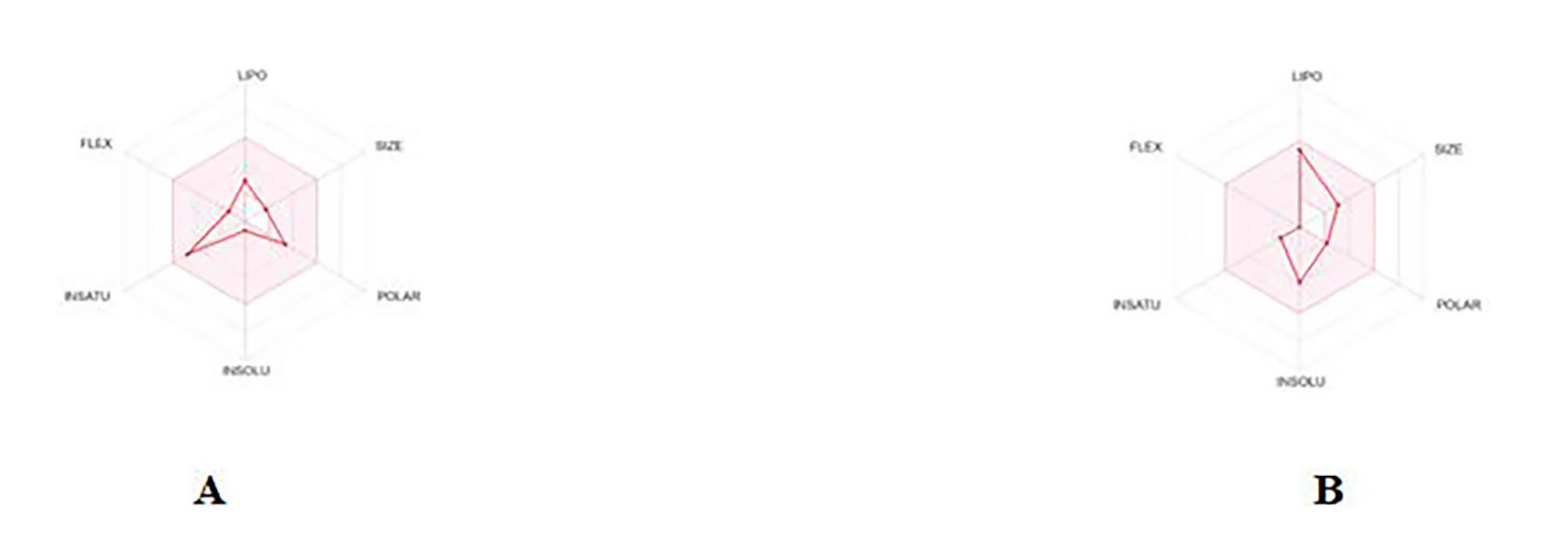
ADME parameters for compound 1 (**A**) and compound 2 (**B**)

To filter the compounds regarding various molecular properties and structural features that is typically associated with successful drugs, the drug likeness of six isolated compounds obtained from the marine sponge *Hemimycale sp.* was assessed using Swiss ADME tool through website http://www.swissadme.ch, this website enables us to calculate physicochemical descriptors and predict ADME parameters, pharmacokinetic properties, drug-like characteristics, and medicinal chemistry suitability for one or multiple small molecules, aiding in drug discovery [22]. The analysis indicated that compounds **1** and **2** obeyed the Lipinski and Veber rules without any violations Compounds that meet Lipinski’s criteria tend to have higher oral bioavailability and better pharmacokinetic profiles while Veber’s rule complements Lipinski’s by accounting for the impact of molecular flexibility and surface polarity, ensuring compounds can pass through cell membranes. (Fig. 3), whereas the remaining compounds did not meet these criteria. Then the potential targets associated with compounds **1** and **2** were identified by analyzing the spatial conformation of these compounds using the Swiss Target Prediction Tools, available at the Swiss Institute of Bioinformatics (https://www.swisstargetprediction.ch/) [23, 24].

#### Protein-protein interaction (PPI) network construction

**Fig. 4:**
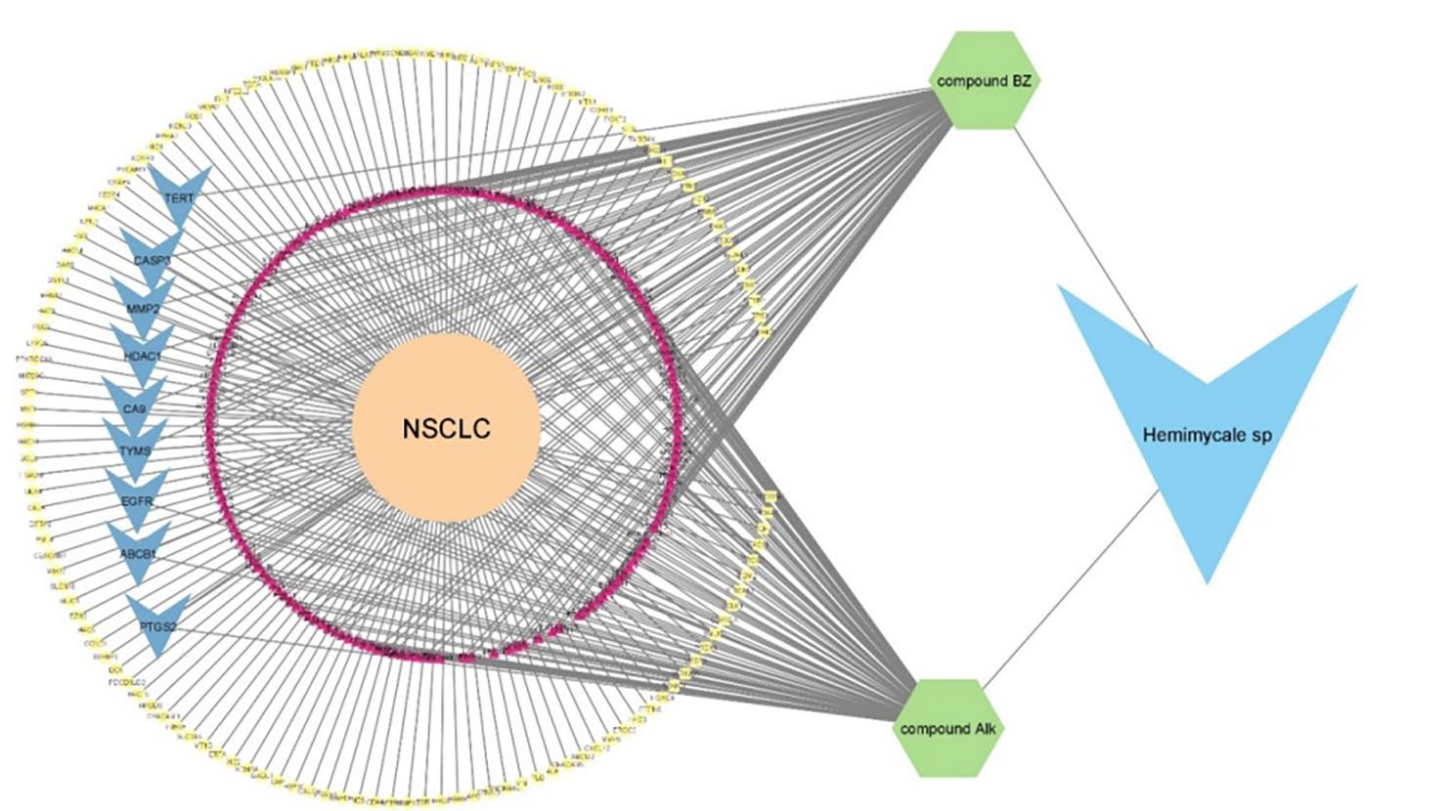
Compounds-disease-targets Network Correspondence of shapes, lines and meanings was as follows: green hexagons correspond to compound; purple triangles correspond to predicted compounds targets; yellow rectangles corresponds to NSCLC targets; blue small V corresponds to intersected targets

**Fig. 5:**
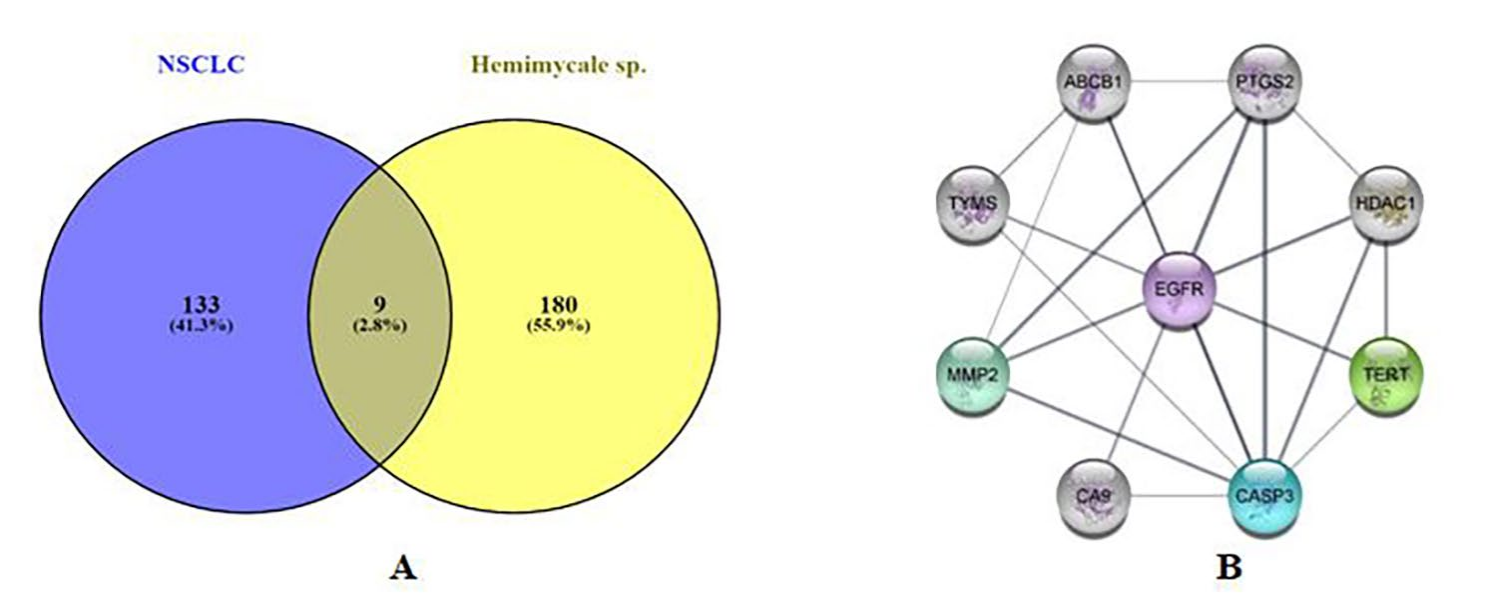
**A**) Venn diagram for compounds **1** and**2** predicted targets and NSCLC genes. **B**) PPI network of the 9 common genes between compounds **1** and **2** targets and NSCLC-regulated genes

The obtained compounds targets were mapped to NSCLC disease targets to obtain a Venn diagram using Venny 2.1 (bioinfogp.cnb.csic.es) [25, 26] of the intersected gene symbols, Subsequently, interactions involving these targets were compiled to construct a comprehensive information network. Finally, a visual analysis of the “Compounds-disease-targets Network” (Fig. 4) was conducted using Cytoscape 3.9.1 software [27, 28]. Cytoscape 3.9.1 is robust bioinformatics software for visualizing molecular interaction networks [29]. The resulted intersected nodes were isolated to be a network composed of 9 nodes with 21 Edges (Fig. 5). The “Compounds-disease-targets Network” visually represents the interactions between bioactive compounds, disease-related targets, and their connections, helping to identify key molecular players in disease pathways. This network aids in understanding the mechanistic relationships and prioritizing drug targets for therapeutic intervention. the analysis of the network showed that EGFR possessed the highest degree of connectivity, betweenness, closeness and radiality, degree of connectivity refers to how many direct connections EGFR has with other nodes, indicating its central role in the network. While betweenness measures how often EGFR lies on the shortest paths between other nodes, suggesting its influence in controlling information flow. Closeness reflects how quickly EGFR can interact with other nodes in the network, highlighting its accessibility within the system, finally radiality assesses how close EGFR is to all other nodes, emphasizing its overall reach and impact on the network. These metrics suggest EGFR’s crucial role in the molecular interactions related to NSCLC disease.

#### Molecular Docking study into EGFR binding site

The targeted compounds were screened against the EGFR catalytic domain by performing molecular docking using the computational program MOE 2019.010. The X-ray crystallographic structure of EGFR catalytic domain in complex with erlotinib was obtained from the Protein Data Bank through the internet (http://www.rcsb.org/pdb/, PDB ID code 4HJO). To validate our study, the ligand was re-docked with the active pocket site of EGFR catalytic domain, the docking algorithm was able to predict the co-crystalized ligand (erlotinib) [30] pose with least RMSD with energy score of **-5.847** kcal/mol showing interactions with receptor in the form of hydrogen binding (HB) with **Lys 721** residue as a H-acceptor and with amino acids **Val 702**,** Cys 773 and Leu 820** as hydrophobic pi-H interaction.

**Fig. 6:**
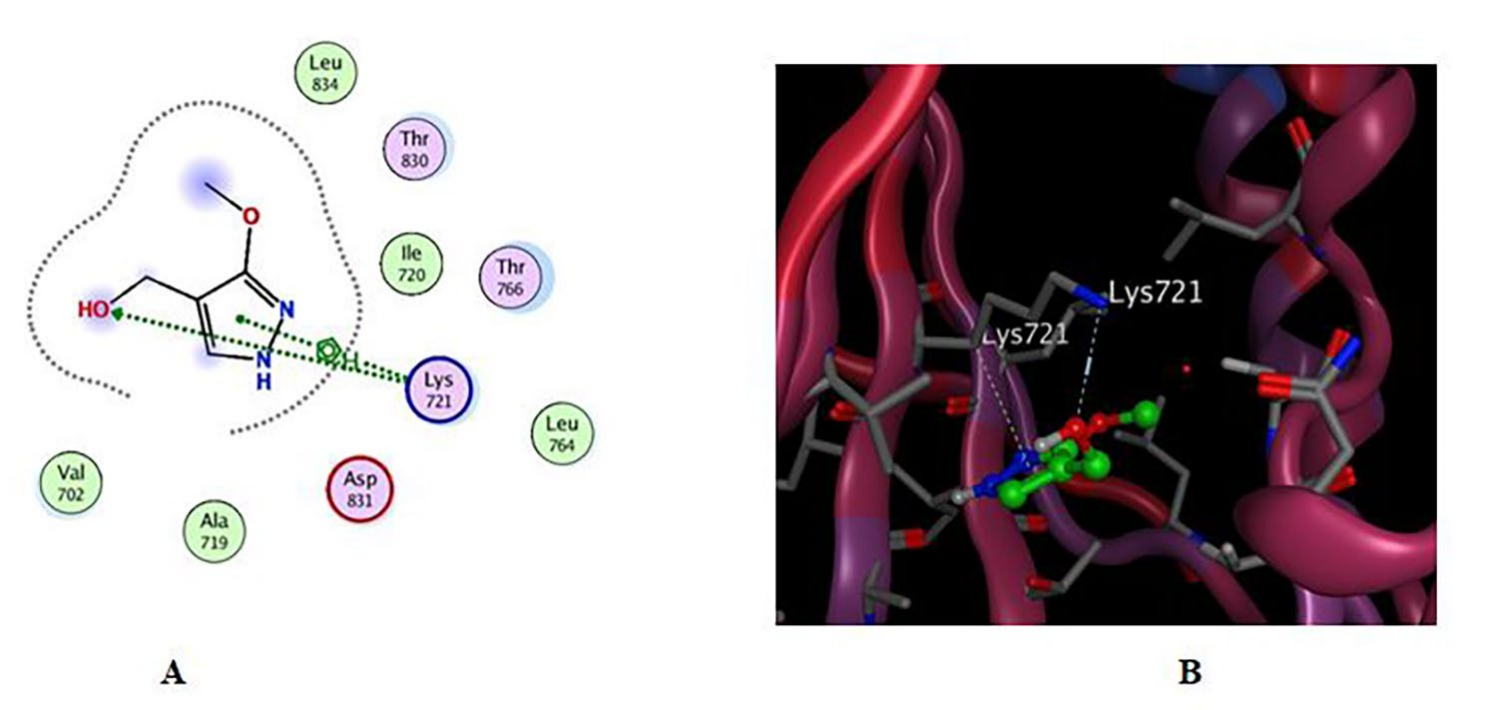
Docking results of compound **1** in the active pocket site of EGFR catalytic domain (4HJO). (**A**) 2D interactions (**B**) Docking pose

**Fig. 7:**
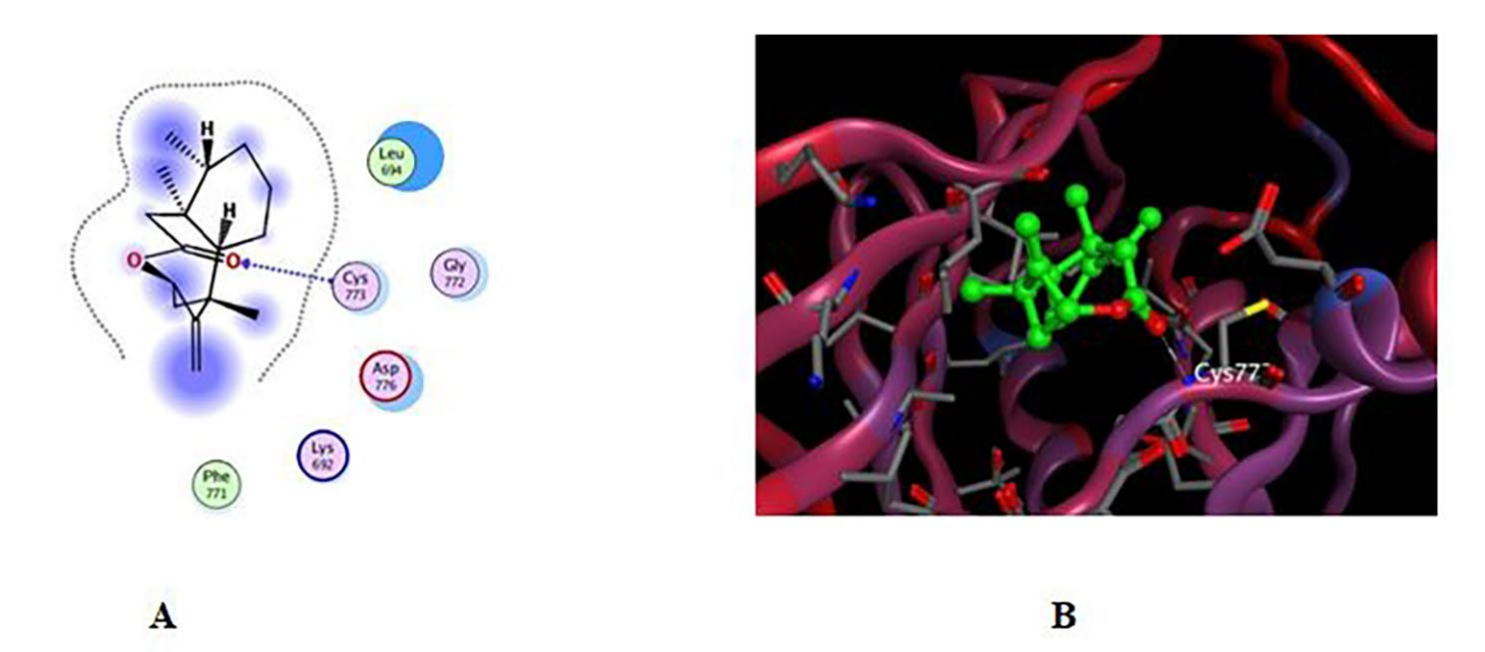
Docking results of compound **2** in the active pocket site of EGFR catalytic domain 4HJO). (**A**) 2D interactions (**B**) Docking pose

Docking results revealed that compounds **1** and **2** both exhibit energy values of -4.789 and − 4.421 kcal/mol, respectively. These values are comparable with erlotinib value of -5.847 kcal/mol. The RMSD_Refineb values for compounds **1** and **2** are 1.29 and 1.61 respectively, which reflect the deviation of the compound’s structure after refinement compared to the reference structure. Regarding interaction mode of the two compounds (Figs. 6 and 7 and Table 3), compound **1** possessed slightly higher affinity towards the EGFR catalytic domain than compound **2**, the 2D interactions shown in (Fig. 6) displayed HB binding between the hydroxymethyl group and amino acid residue Lys 721 as H-acceptor at distance of 3.12 A֯ which resembles the ligand mode of interaction in addition to pi-H interaction between the pi system in the pyrazolyl moiety with the same residue, while compound **2** showed only one interaction through the carbonyl moiety with Cys 773 as H-acceptor at distance of 3.07 A֯ the same amino acid residue form an interaction with erlotinib but in form of pi-H interaction. In conclusion, the molecular docking approach exhibited that the hydroxy methyl moiety, hydrophobic fragment in **compound 1**, and the carbonyl group at **compound 2** can act as hydrogen bond acceptors to generate hydrogen bond interactions as represented in Table 3 [31].

**Table 3:** Receptor interactions and binding energies of the compound X and erlotinib into the active pocket site of EGFR catalytic domain

NO.	Compound	S_a_ kcal/mol	RMSD_Refine_b_	Amino acid bond	Distance A֯	E (Kcal mol)
1	Compound **1**	-4.789	1.29	LYS 721 / H-acceptor	3.12	-3.3
				LYS 721 / pi-H	3.65	-0.5
2	Compound **2**	-4.421	1.61	CYS 773 / H-acceptor	3.07	-3.3
3	erlotinib	-5.847	1.53	Lys 721/H-acceptor	3.53	-2
				Val 702/ pi-H	3.82	-0.8
				Cys 773 / pi-H	3.98	-0.5
				Leu 820 /pi-H	3.83	-0.6

^a^ S: the score of a compound placement inside the protein binding pocket

^b^ RMSD_Refine: the root-mean-squared-deviation (RMSD) between the predicted pose and those of the crystal one (after and before refinement process, respectively)

## Experimental

### General experimental procedures

^1^H, ^13^C, Dept-135 and 2D NMR data were measured by a Bruker AVANCE spectrometer at 500 MHz and 125 MHz, (Bruker, Germany) and also Bruker BioSpin GmbH spectrometer at 400 MHzand 100 MHz (Bruker, Germany), respectively. HR-ESI-MS data were recorded through a Thermo Fisher Scientific LTQ Orbitrap XL spectrometer. A source of high-emission low-energy electron ionization was conducted with electron energy of 70 eV and an emission current of 25.0 µA was fitted to The Agilent 7250 Accurate-Mass Quadrupole Time-of-Flight (Q-ToF) mass spectrometer. The temperatures of source, quadrupole, and transfer line were 230 °C, 150 °C, and 280 °C, respectively throughout the experiment. As, the mass spectral data have been obtained at a rate of 5 Hz from 40 to 450 *m/z* after a delay of seven min solvent (SCIEX, US). A P-1030 spectropolarimeter (JASCO, Tokyo, Japan) was used for the measurement of the specific optical rotation. IR spectrum was measured on FT/IR-4600 (JASCO, Kyoto, Japan). Precoated silica gel 60 F254 plates (E. Merck, Germany) were used for TLC detection. Silica gel stationary phase for column chromatography (60–120 mesh, E. Merck, Germany) and Sephadex LH-20 stationary phase (GE Health Care, Sweden) were used for chromatographic separation of the compounds. The cancer cell line A549 was acquired from Sigma-Aldrich, Germany. All solvents were acquired from Merck (Germany) and Sigma Chemical Co. (USA), and were of analytical grades.

### Cytotoxic assay

The cytotoxic activity of different fractions; petroleum ether (A1), 50% petroleum ether in ethyl acetate (A2), ethyl acetate (A3), 50% ethyl acetate in methanol (A4) and methanol (A5) was evaluated by MTT assay against lung cancer cell line (A549). In this experiment, 96-well tissue culture plates were seeded with cells at a concentration of 1 × 10^5^ cells/mL, with 100 µL of the cell suspension added to each well. The plates were incubated at 37 °C for 24 h to allow the cells to form a complete monolayer. Once confluence was achieved, the growth medium was carefully removed, and the cell monolayers were washed twice with a wash medium. Serial two-fold dilutions of the test sample were then prepared in RPMI medium supplemented with 2% serum (maintenance medium). A volume of 0.1 mL from each dilution was added to the corresponding wells, while three wells served as controls and received only the maintenance medium. The plates were incubated again at 37 °C and monitored for signs of cytotoxicity, such as the partial or complete disruption of the monolayer, cell rounding, shrinkage, or granulation. Next, an MTT solution (5 mg/ml in PBS) from BIO BASIC CANADA INC was prepared and 20 µl of this solution was added to each well. The plates were shaken at 150 rpm for 5 minutes to ensure thorough mixing, followed by incubation at 37 °C with 5% CO_2_ for 1 to 5 hours to allow the MTT to be metabolized. After incubation, the media was carefully removed to eliminate any remaining residues, and the formazan crystals (the MTT metabolic product) were dissolved in 200 µL of DMSO. The plates were shaken again at 150 rpm for 5 minutes to mix the formazan thoroughly. Finally, the optical density was measured at 560 nm, with a background subtraction at 620 nm, to correlate the absorbance with the cell quantity in each well [15].

### Marine sponge material

Sponge material was collected from a long patchy reef named Ahia Reefs, Hurghada and identified by Dr. El-Sayed Abd El-Aziz (Department of Invertebrates Lab., National Institute of Oceanography and Fisheries, Red Sea Branch, 84511 Hurghada, Egypt) as mentioned in a previous work [16].

### Extraction and isolation of compounds

The sponge was cut into pieces; and extracted with methanol. The methanolic extract was fractionated by vacuum liquid chromatography using different polarities to give five major fractions (A1-A5) as mentioned before [16]. The ethyl acetate fraction (A3) was subjected to sequential fractionation on silica gel column chromatography using CH_2_Cl_2_-MeOH in gradient elution manner yielding six subfractions (sb1-sb6). Subfraction sb2 was subjected to further purification on silica gel column chromatography and sephadex LH-20 stationary phases using CH_2_Cl_2_-MeOH in gradient elution manner and methanol in isocratic elution, respectively, which lead to isolation of compounds **1** (3 mg) which was isolated as yellowish white amorphous powder and **2** (2 mg) isolated as white amorphous powder. Compounds **3**–**6** were isolated from 50% petroleum ether in ethyl acetate fraction (A2) by successive fractionation and purification on silica gel column chromatography using petroleum ether – ethyl acetate mixture in gradient elution manner.

### Construction of pharmacological network

#### Construction of NSCLC-Targets network

NSCLC-related genes were identified using DisGeNET, querying the database for “Adenocarcinoma of lung disorder,” resulting in 2438 associated genes. The identified genes were analyzed with HumanNet, Tissue gene expression data from BioGPS was used to refine the analysis.

#### Construction of compound Target Network

Six compounds isolated from the marine sponge *Hemimycale* sp. were evaluated for drug-likeness using the Swiss ADME tool. Physicochemical descriptors, ADME parameters, and drug-like characteristics were calculated. Potential targets for compounds **1** and **2** were predicted by analyzing their spatial conformations using the Swiss Target Prediction tool.

#### Protein-protein Interaction (PPI) Network Construction

Targets of the obtained compounds were mapped to non-small cell lung cancer (NSCLC) disease targets, generating a Venn diagram using Venny 2.1. Interactions between these intersected targets were compiled to form a network, which was visualized using Cytoscape 3.9.1 software. Network analysis identified EGFR as the node with the highest degree of connectivity, betweenness, closeness, and radiality.

### Molecular Docking study into EGFR binding site

The X-ray crystallographic structure of protein target EGFR catalytic domain in complex with erlotinib was obtained from the Protein Data Bank through the internet (http://www.rcsb.org/pdb/, code 4HJO) All molecular modelling calculations and docking studies were carried out using ‘Molecular Operating Environment 2019.0102 software (MOE). Validation of docking approach was performed by redocking process of the original ligand (erlotinib) into the binding sites of selected protein [30, 32]. The preparation of the protein included the removal of water molecules and using the quick preparation tool in MOE with applying the default options. Docking of the conformation database of the target compounds was done after preparation of the enzyme. The following methodology was generally applied: the enzyme active site was located by the site finder tool, and the docking tool was initiated. The program specifications were adjusted to ligand atoms as the docking site, alpha triangle as the placement methodology to be used. The scoring methodology London dG is used and was adjusted to its default values. The MDB file of the ligand to be docked was loaded and dock calculations were run automatically [33]. Receptor-ligand interactions of the complexes were examined in 2D and 3D styles the poses that showed best ligand–enzyme interactions were selected and stored for energy calculations. The selection of poses was done according to their better obtained binding scores and RMSD Refine values, the obtained scores, RMSD Refine values, and interactions with binding pocket site of the enzymes are discussed [27, 34].

## Conclusion

In this study, two novel compounds, 4-(hydroxymethyl)-3-methoxy-1 H-pyrazole and mycalene, along with four known metabolites, were successfully isolated from the ethyl acetate and petroleum ether : ethyl acetate (1:1) fractions of the Red Sea marine sponge *Hemimycale* sp., which demonstrated cytotoxic activity against lung cancer cell line (A549). Network analysis revealed potential interactions between compounds **1**, **2** and known NSCLC targets, with EGFR (Epidermal Growth Factor Receptor) showing the highest degree of connectivity. Molecular docking simulations demonstrated favourable binding energies for compounds **1** and **2** within the EGFR catalytic domain, suggesting their potential as EGFR inhibitors. These findings warrant further investigation of these novel compounds for their potential as therapeutic leads for lung cancer. Both in vitro and in vivo studies are needed to validate the anti-cancer activity of compounds **1** and **2.** Mechanistic studies should also be conducted to elucidate the specific molecular pathways targeted by these compounds. Further structural optimization may enhance their potency and selectivity. Overall, this study provides promising preliminary data, supporting the potential development of novel therapeutics derived from marine sponges for lung cancer treatment.

## Electronic supplementary material

Below is the link to the electronic supplementary material.

Supplementary Material 1

## Data Availability

Data is provided within the manuscript or supplementary information files.
