# Supplementary material for: Bioassay-guided isolation and in Silico characterization of cytotoxic compounds from Hemimycale sp. Sponge targeting A549 lung cancer cells
Source: BMC Chem. 2024 Nov 1;18(1):213. doi: 10.1186/s13065-024-01325-w (PMC11531136; doi:10.1186/s13065-024-01325-w)
Supplement: Supplementary file 1 — Supplementary Material 1 [file 13065_2024_1325_MOESM1_ESM.docx]

**Bioassay-Guided Isolation and *In Silico* Characterization of Cytotoxic Compounds from *Hemimycale* sp. Sponge Targeting A549 Lung Cancer Cells**

Asmaa Abo Elgoud Said^a,^, Islam M. Abdel-Rahman^b^, Yasser A. Mostafa^c^, Eman Zekry Attia^a,^ , Mamdouh Nabil Samy^a^, Usama Ramadan Abdelmohsen^a,d*^, Katsuyoshi Matsunami^e^ , Mostafa A. Fouad^a^, Yaser G. Gouda^f^

^a^ Department of Pharmacognosy, Faculty of Pharmacy, Minia University, 61519 Minia, Egypt.
^b^ Department of Pharmaceutical Chemistry, Faculty of Pharmacy, Deraya University, New-Minia 61111, Egypt

^c^ Pharmaceutical Organic Chemistry Department, Faculty of Pharmacy, Assiut University, 71526 Assiut, Egypt

^d^ Department of Pharmacognosy, Faculty of Pharmacy, Deraya University, Universities Zone, 61111 New Minia City, Egypt.

^e^ Graduate School of Biomedical and Health Sciences, Hiroshima University; 1–2–3 Kasumi, Minami-ku, Hiroshima 734–8553, Japan.

^f^ Department of Pharmacognosy, Faculty of Pharmacy, Assiut University, 71526 Assiut, Egypt

*Correspondence: usama.ramadan@mu.edu.eg (U.R.A.)

| Table of Contents: | Page |
| --- | --- |
| Figure **S1**: ^1^H-NMR spectral data of compound 1 (500 MHz, CDCl_3_). | **S3** |
| Figure **S2**: ^13^C spectral data of compound 1 (125 MHz, CDCl_3_). | **S3** |
| Figure **S3**: Negative HR-ESI-MS spectrum of compound 1 | **S4** |
| Figure **S4**: ^1^H-NMR spectral data of compound 2 (500 MHz, CDCl_3_). | **S4** |
| Figure **S5**: Expanded ^1^H-NMR spectral data of compound 2 (500 MHz, CDCl_3_). | **S5** |
| Figure **S6**: ^13^C spectral data of compound 2 (125 MHz, CDCl_3_). | **S5** |
| Figure **S7**: Expanded ^13^C spectral data of compound 2 (125 MHz, CDCl_3_). | **S6** |
| Figure **S8**: DEPT spectral data of compound 2 (125 MHz, CDCl_3_). | **S6** |
| Figure **S9**: ^1^H-^1^H COSY spectrum of compound 2 | **S7** |
| Figure **S10**: HSQC spectrum of compound 2 | **S7** |
| Figure **S11**: HMBC spectrum of compound 2 | **S8** |
| Figure **S12**: NOESY spectrum of compound 2 | **S8** |
| Figure **S13**: Positive HR-ESI-MS spectrum of compound 2 | **S9** |
| Figure **S14**: FTIR spectrum of compound 2 | **S10** |
| Figure **S15**: ^1^H-NMR spectral data of compound 3 (400 MH_z_, pyridine-*d*_5_) | **S11** |
| Figure **S16**: DEPT-Q spectrum of compound 3 (100 MH_z_, pyridine-*d*_5_) | **S11** |
| Figure **S17**: EI-MS spectrum of compound 3 | **S12** |
| Figure **S18**: Fragmentation pattern of compound 3 | **S12** |
| Figure **S19**: ^1^H-NMR spectral data of compound 4 (500 MHz, CDCl_3_). | **S13** |
| Figure **S20**: ^13^C spectral data of compound 4 (125 MHz, CDCl_3_). | **S13** |
| Figure **S21**: DEPT spectral data of compound 4 (125 MHz, CDCl_3_). | **S14** |
| Figure **S22**: Positive HR-ESI-MS spectrum of compound 4 | **S14** |
| Figure **S23**: ^1^H-NMR spectral data of compound 5 (500 MHz, CDCl_3_). | **S15** |
| Figure **S24**: Positive HR-ESI-MS spectrum of compound 5 | **S15** |
| Figure **S25**: ^1^H-NMR spectral data of compound 6 (500 MHz, CDCl_3_). | **S16** |
| Figure **S26**: ^13^C spectral data of compound 6 (125 MHz, CDCl_3_). | **S16** |
| Figure **S27**: Positive HR-ESI-MS spectrum of compound 6 | **S17** |
| Table **S1:** Cytotoxic activity of different fractions against lung cancer cell line | **S18** |


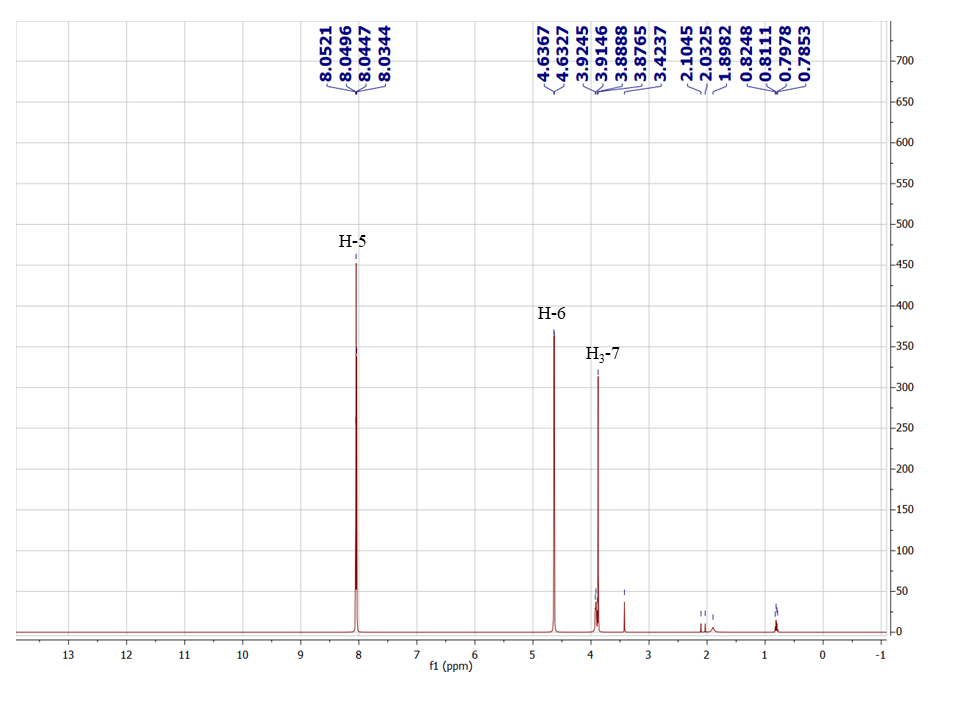


Figure **S1**: ^1^H-NMR spectral data of compound 1 (500 MHz, CDCl_3_).


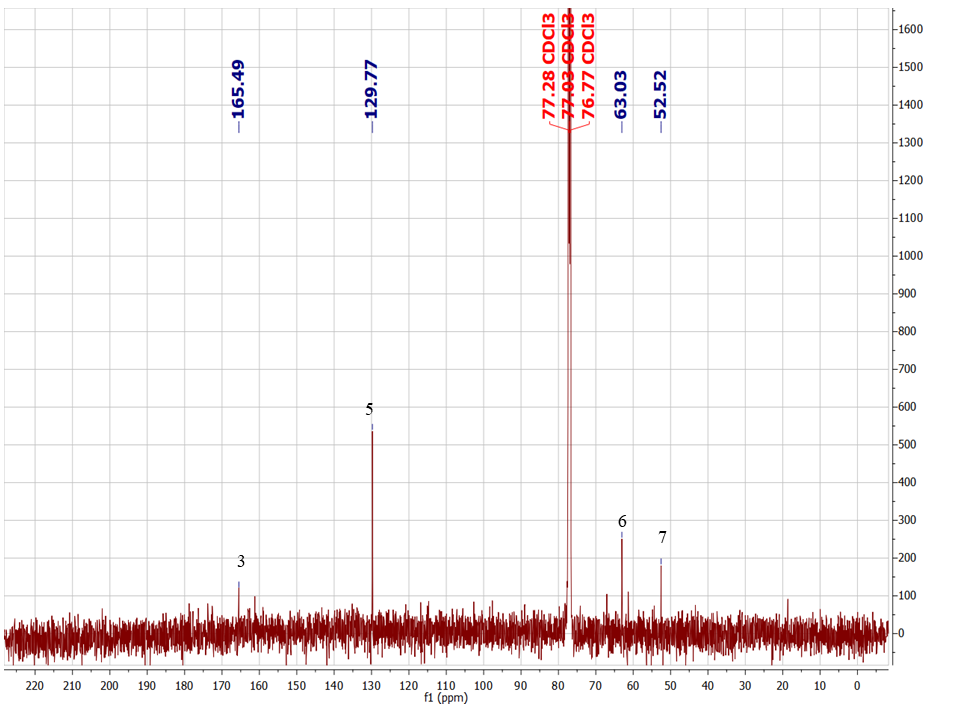


Figure **S2**: ^13^C spectral data of compound 1 (125 MHz, CDCl_3_).


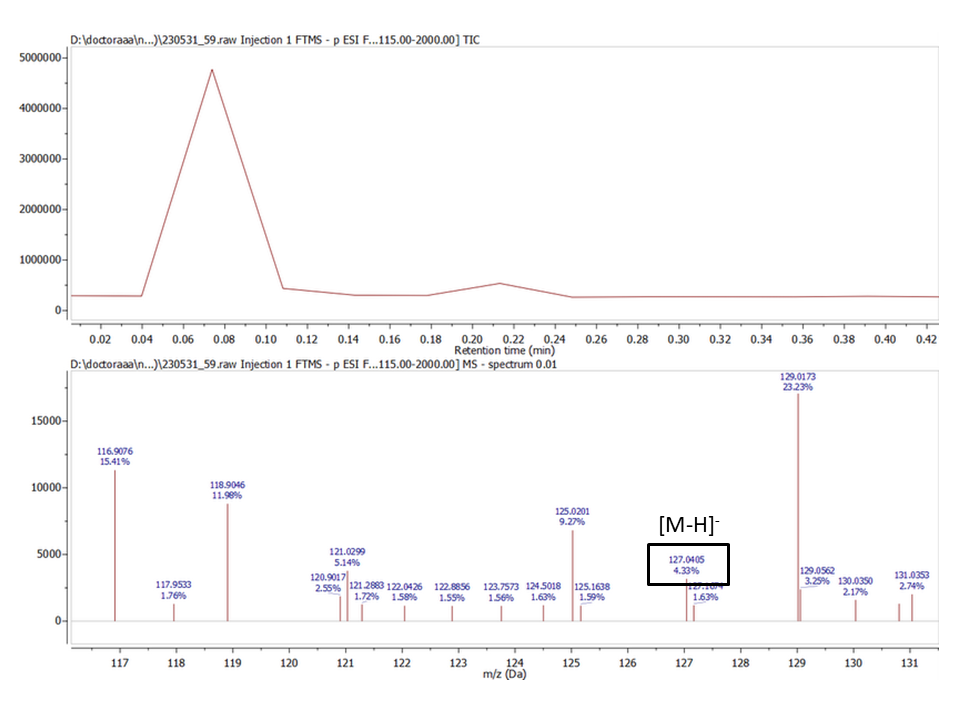


Figure **S3**: Negative HR-ESI-MS spectrum of compound 1


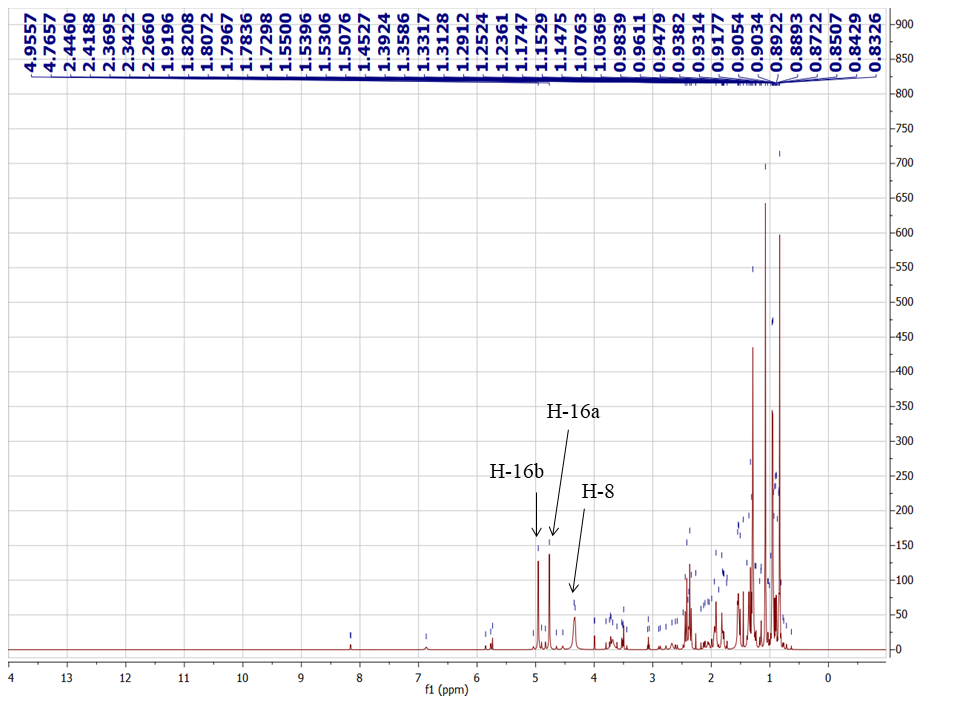


Figure **S4**: ^1^H-NMR spectral data of compound 2 (500 MHz, CDCl_3_).


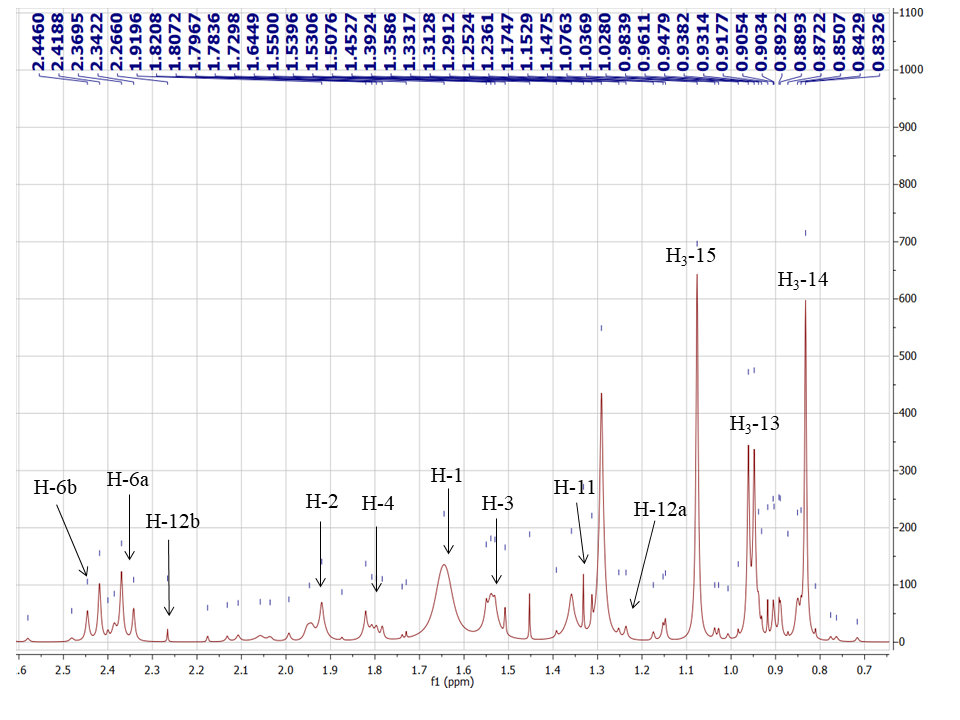


Figure **S5**: Expanded ^1^H-NMR spectral data of compound 2 (500 MHz, CDCl_3_).


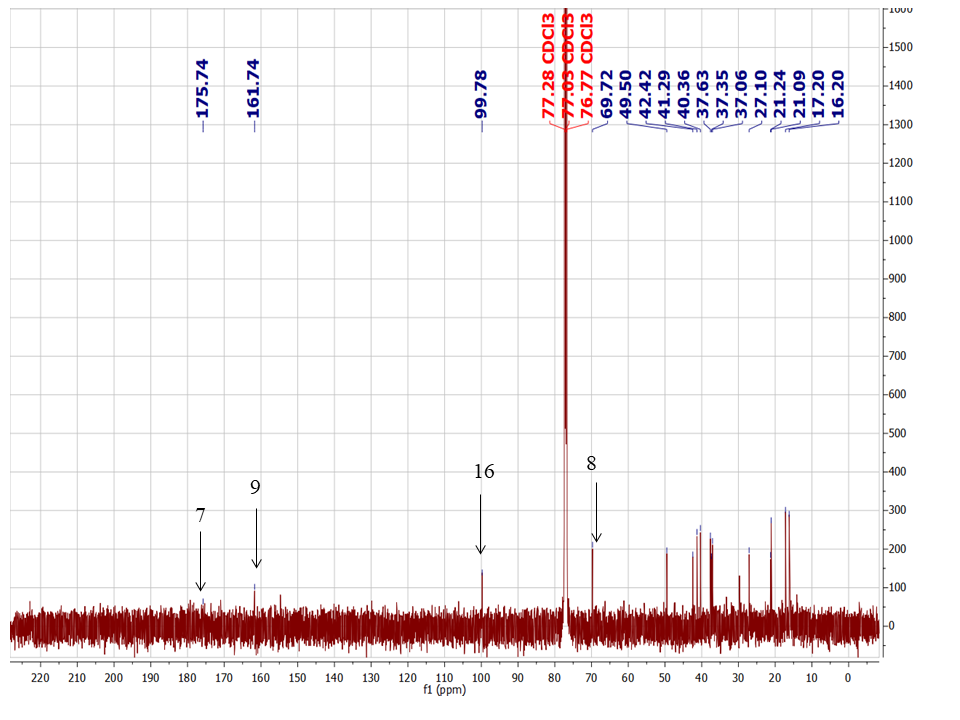


Figure **S6**: ^13^C spectral data of compound 2 (125 MHz, CDCl_3_).


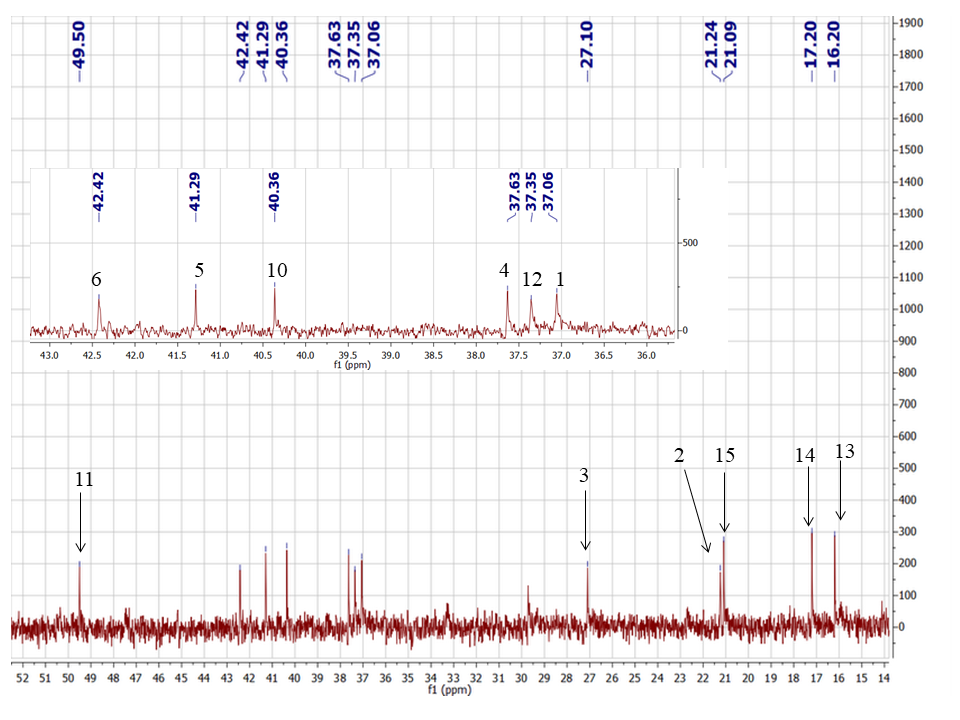


Figure **S7**: Expanded ^13^C spectral data of compound 2 (125 MHz, CDCl_3_).


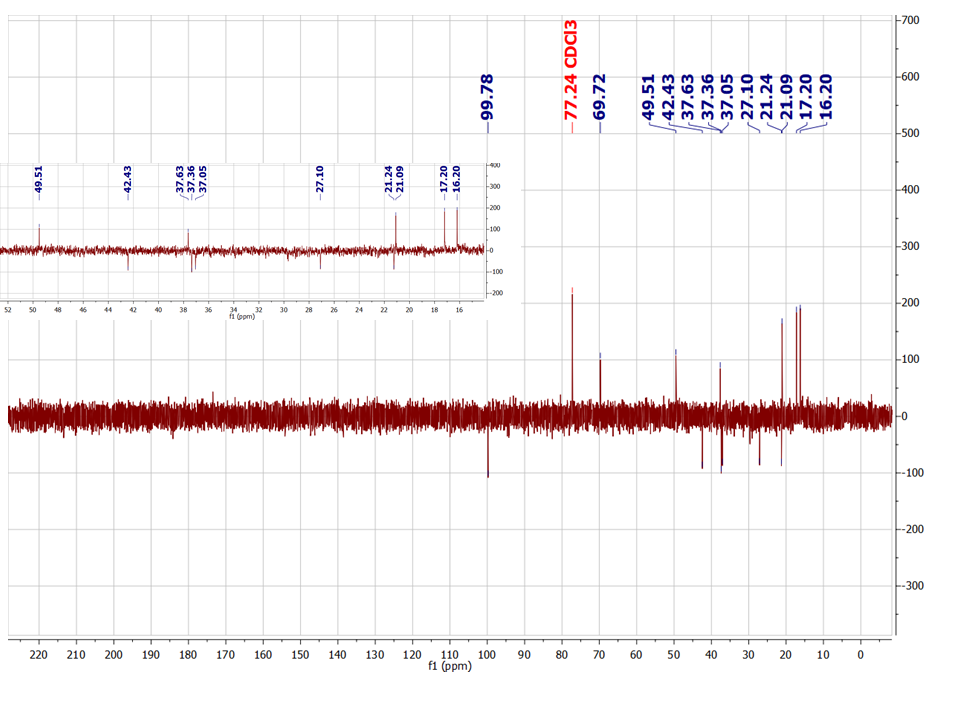


Figure **S8**: Dept-135 spectral data of compound 2 (125 MHz, CDCl_3_).


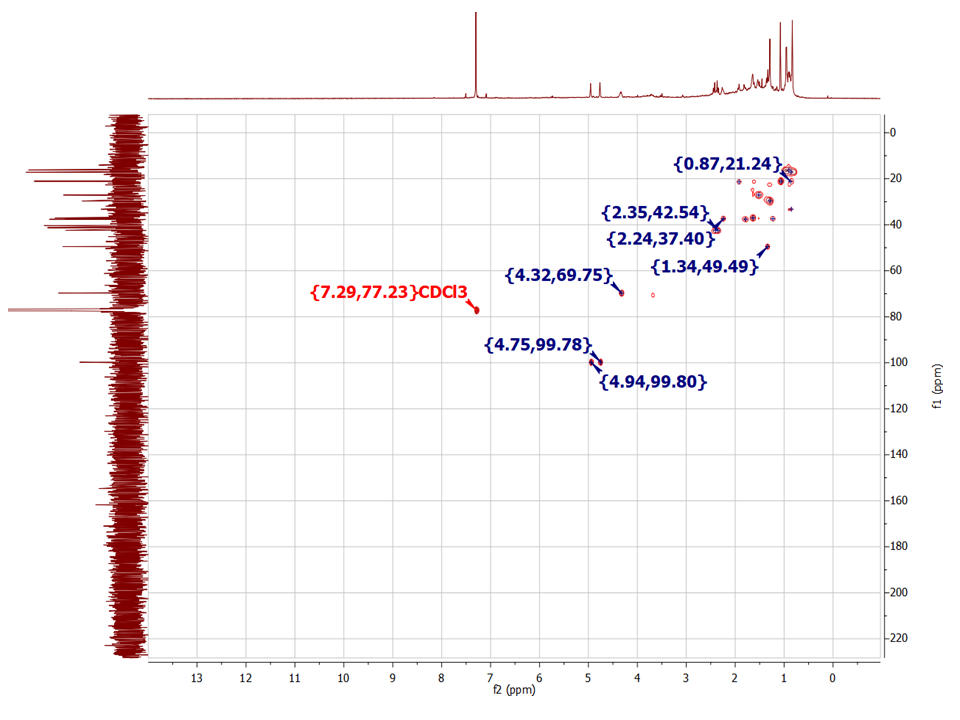


Figure **S9**: HSQC spectral data of compound 2 (125 MHz, CDCl_3_).


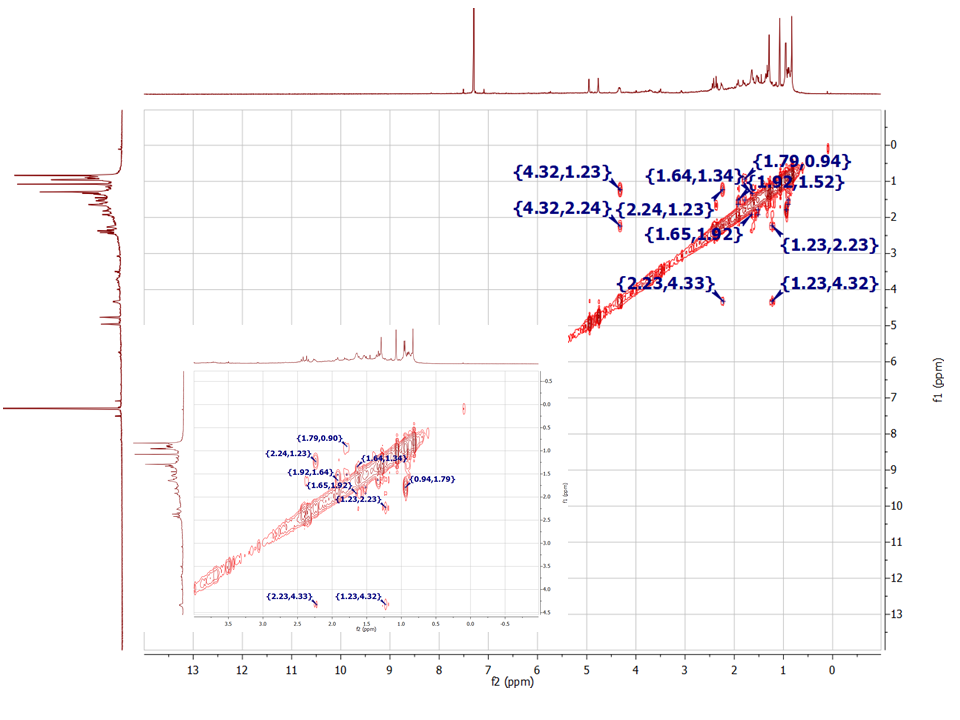


Figure **S10**: ^1^H-^1^H COSY spectrum of compound 2.


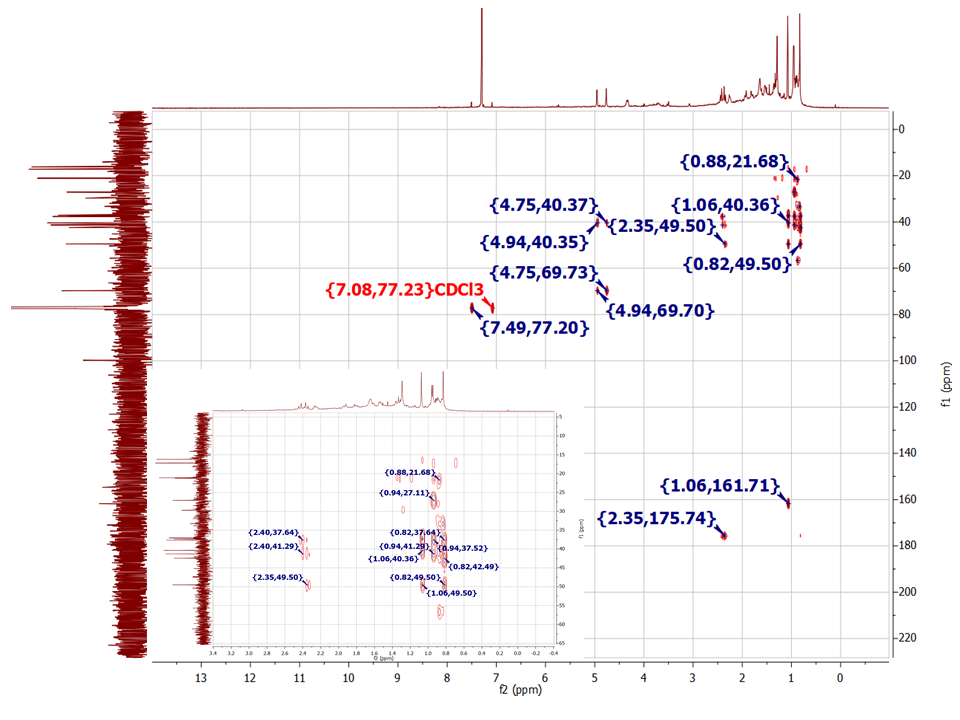


Figure **S11**: HMBC spectrum of compound 2


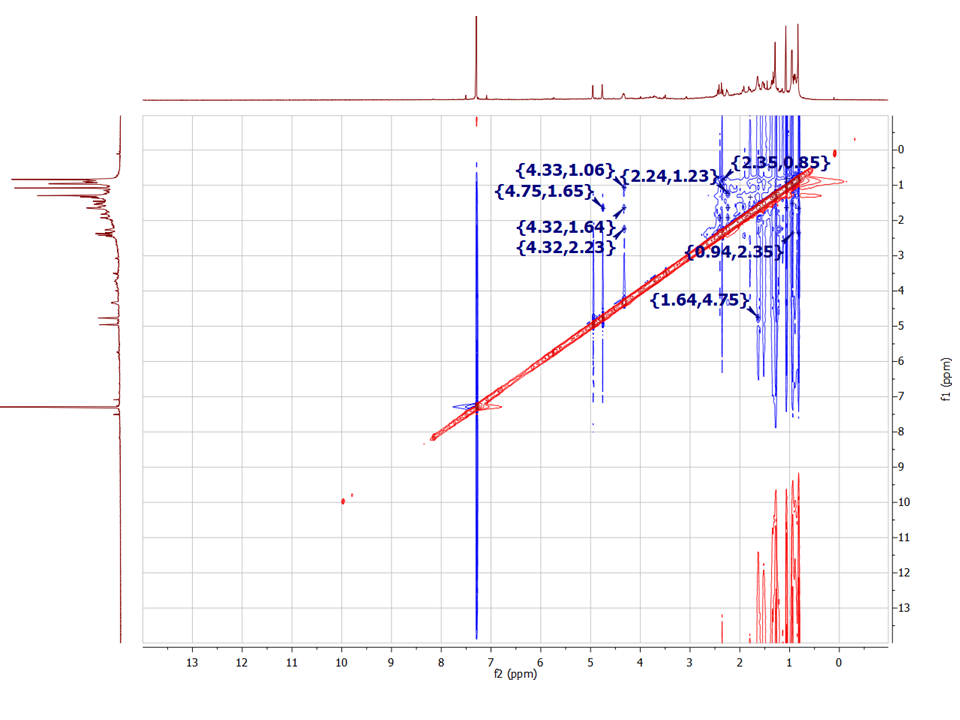


Figure **S12**: NOESY spectrum of compound **2** (500 MH_Z_, CDCl_3_).


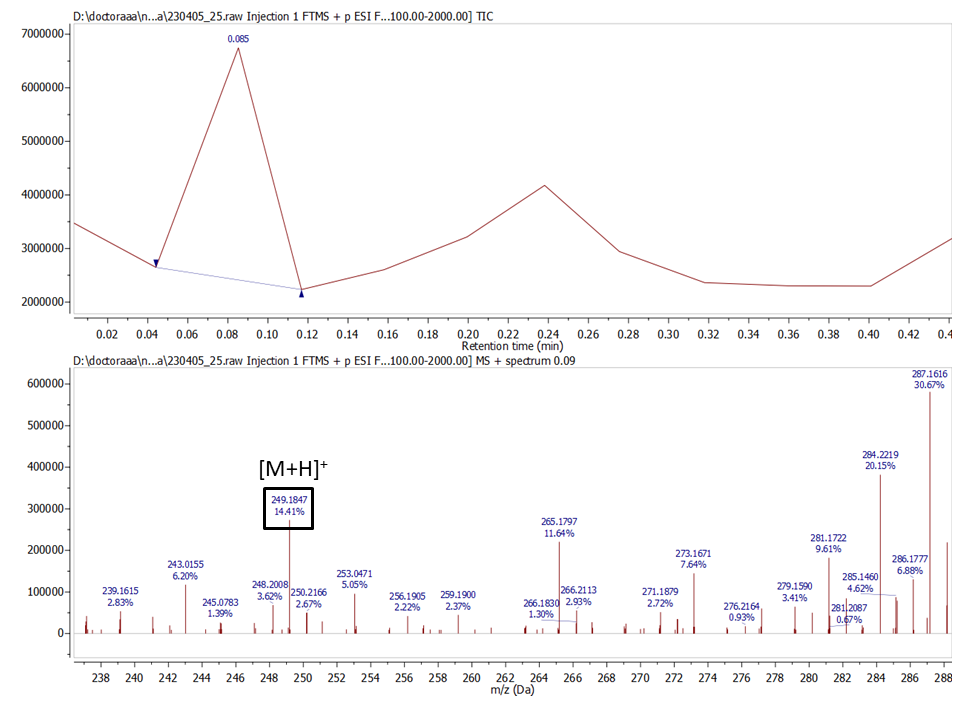


Figure **S13**: Positive HR-ESI-MS spectrum of compound **2.**

Figure **S14**: FTIR spectrum of compound **2.**


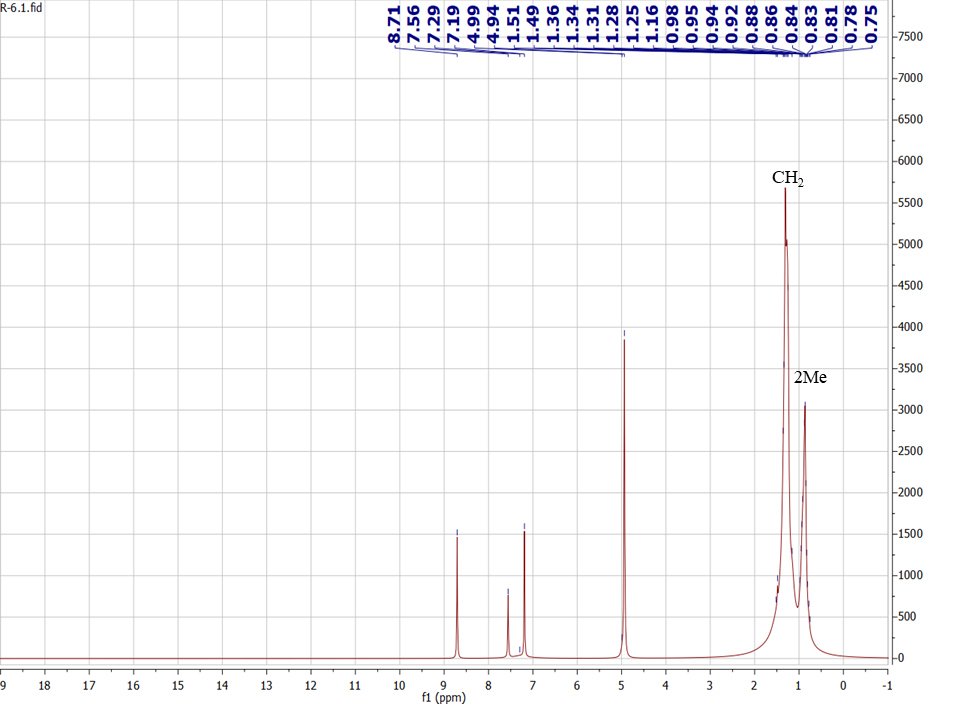


Figure **S15**: ^1^H NMR spectrum of compound **3** (400 MH_Z_, pyridine-*d_5_*).


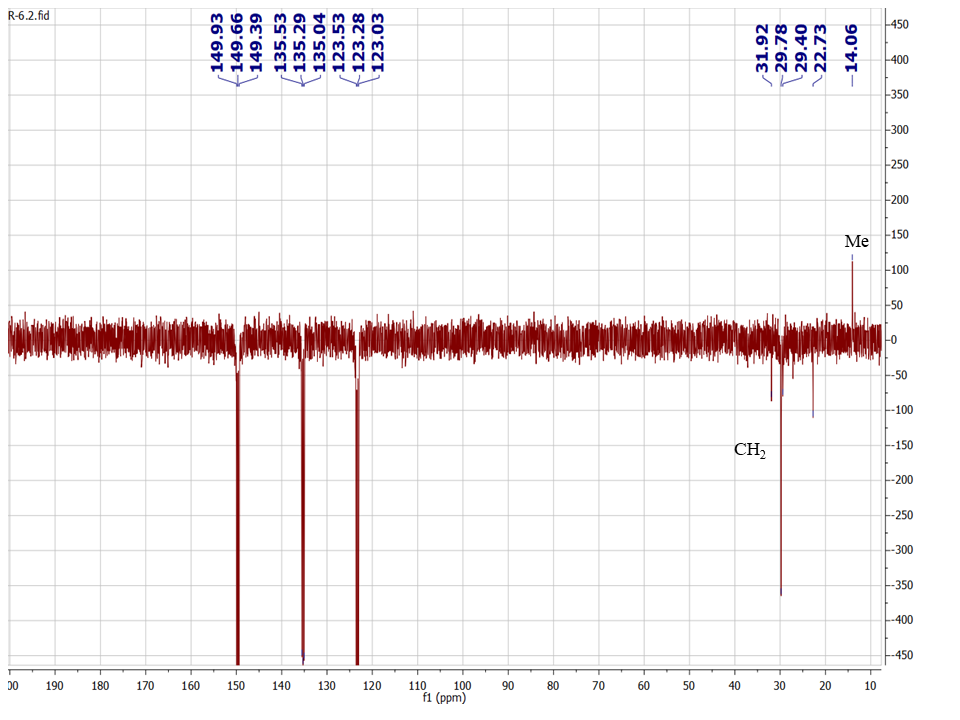


Figure **S16**: DEPT-Q NMR spectrum of compound **3** (100 MH_Z_, pyridine-*d_5_*).


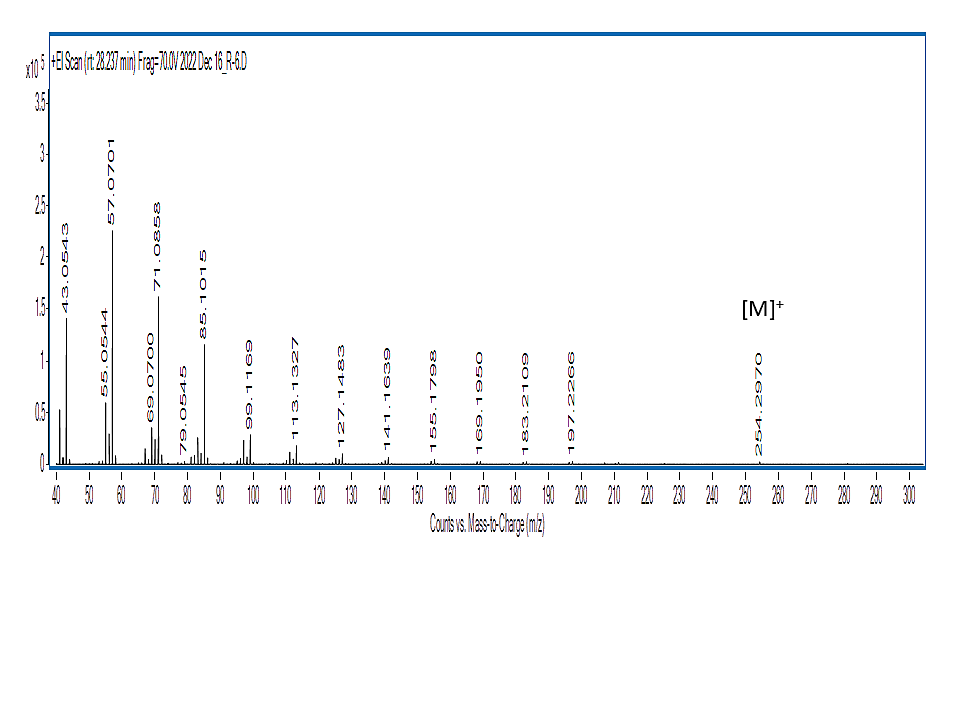


Figure **S17**: EI-MS spectrum of compound **3.**

Figure **S18**: Fragmentation pattern of compound **3.**


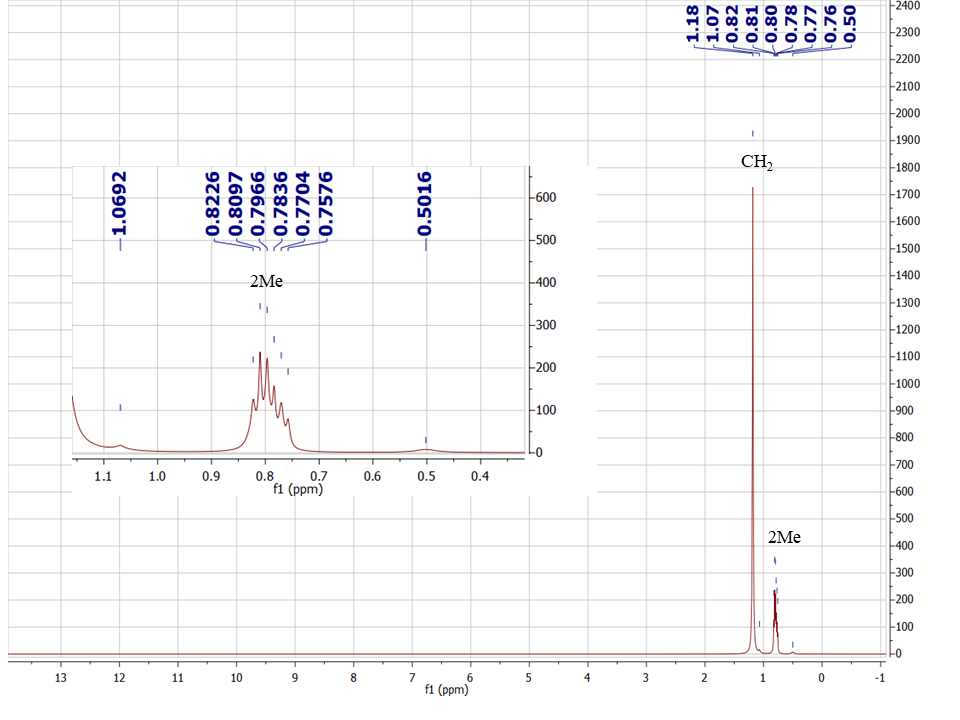


Figure **S19**: ^1^H-NMR spectral data of compound 4 (500 MHz, CDCl_3_).


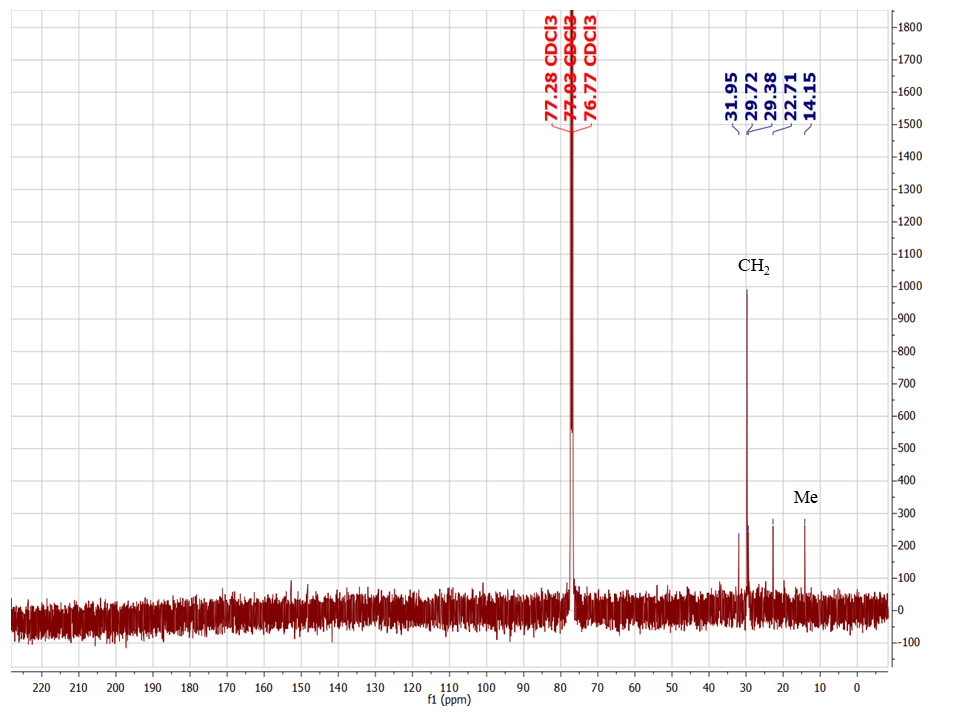


Figure **S20**: ^13^C spectral data of compound 4 (125 MHz, CDCl_3_).


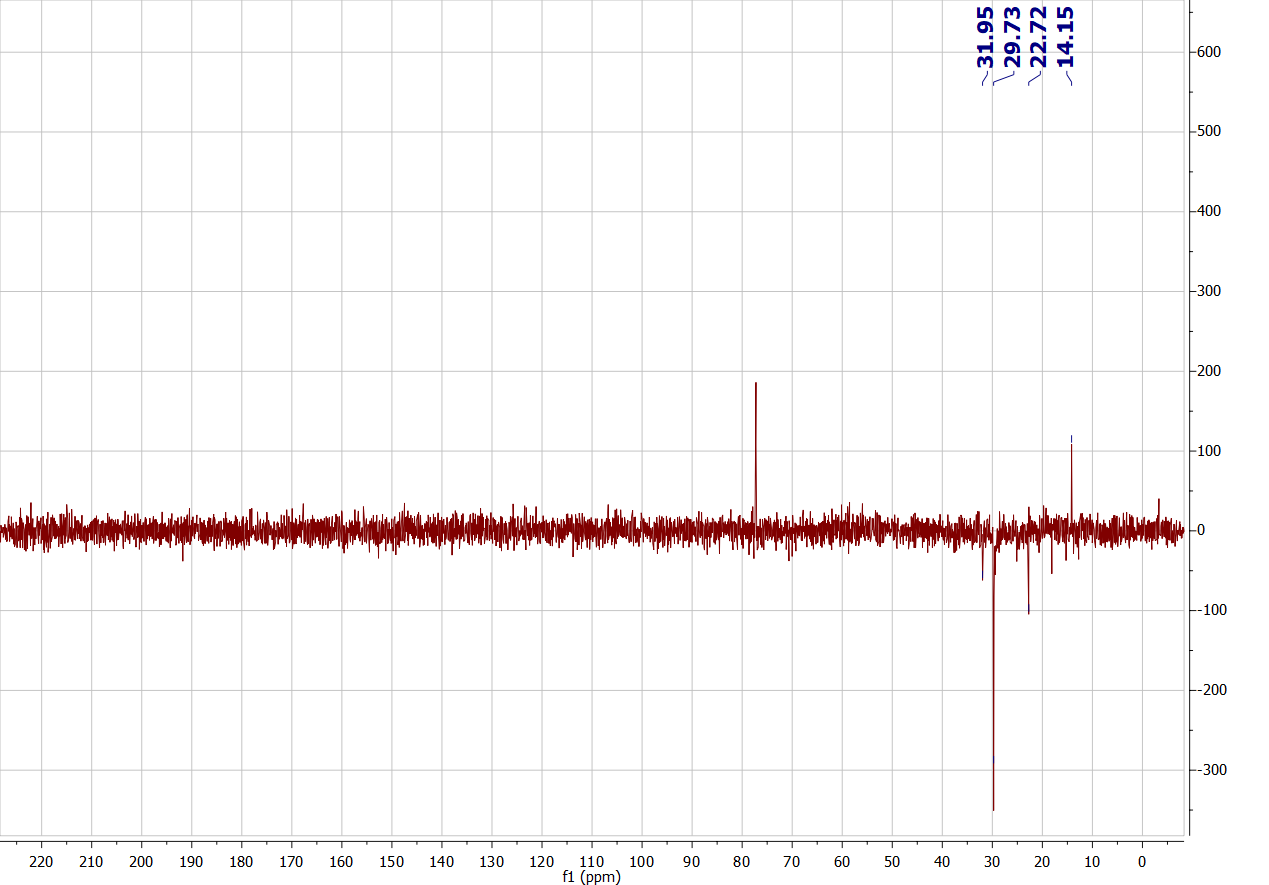


Figure **S21**: DEPT-135 spectral data of compound 4 (125 MHz, CDCl_3_).


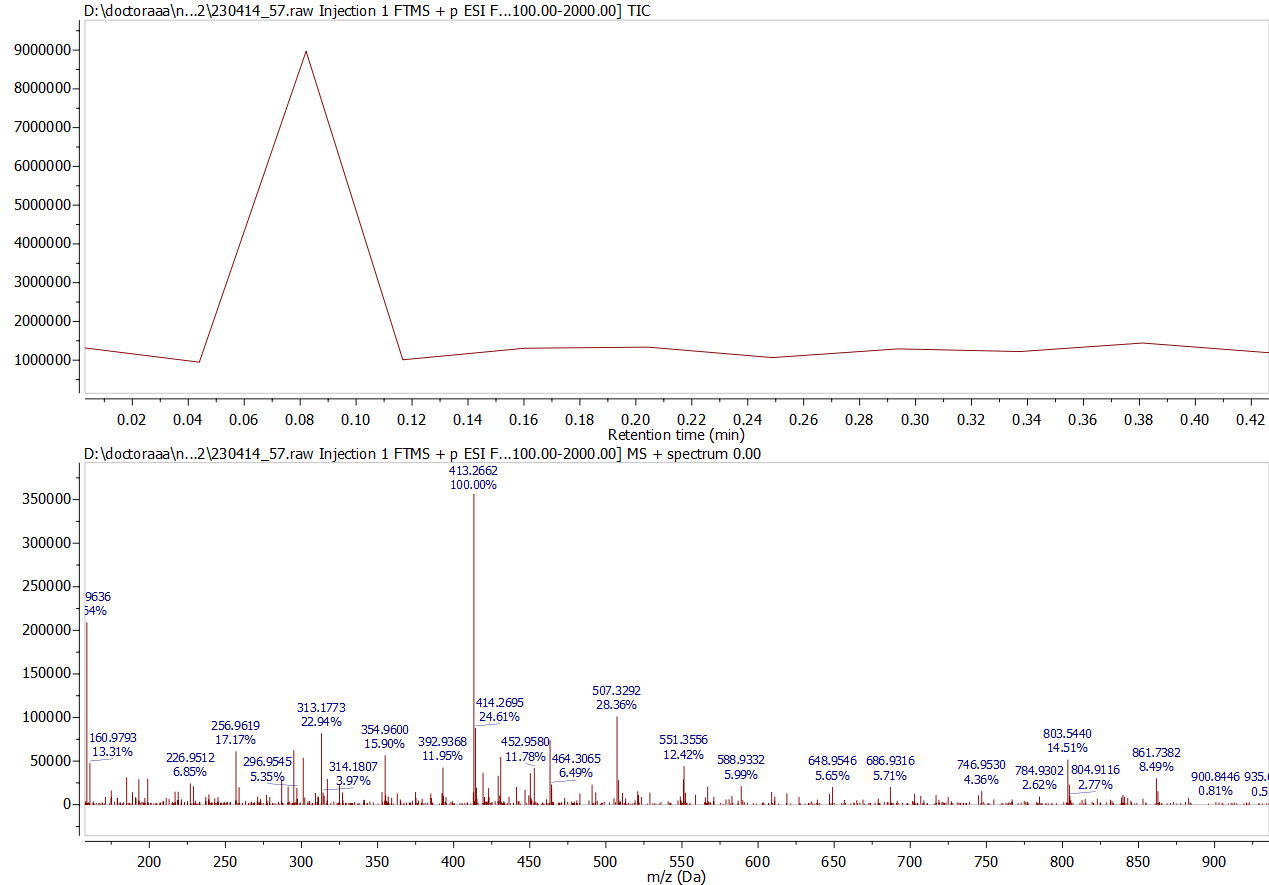


[M+H]^+^

Figure **S22**: Positive HR-ESI-MS spectrum of compound 4.


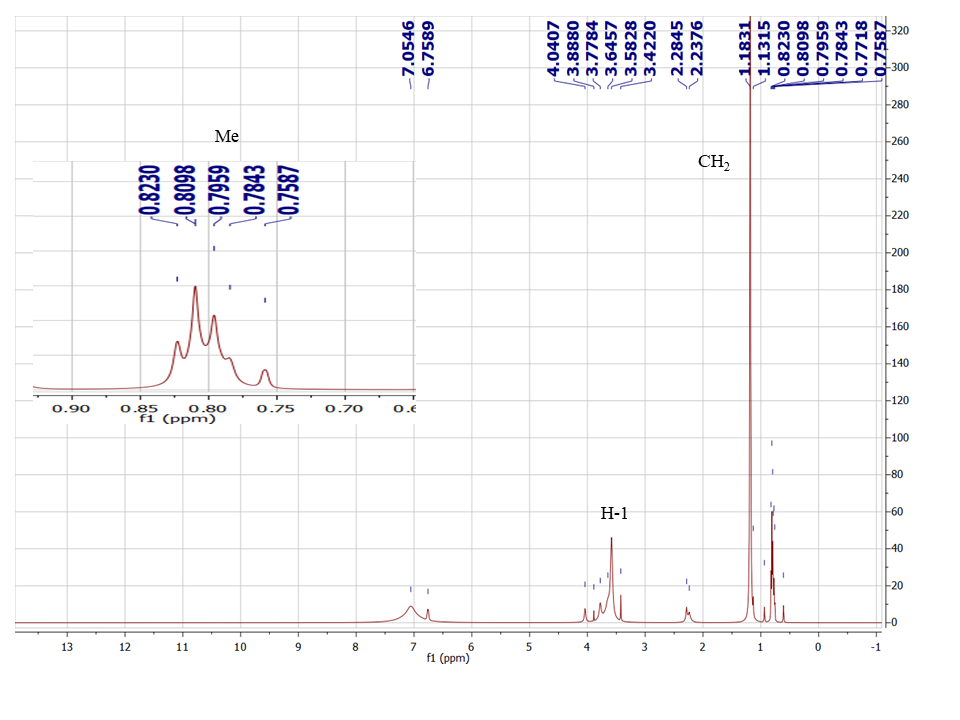


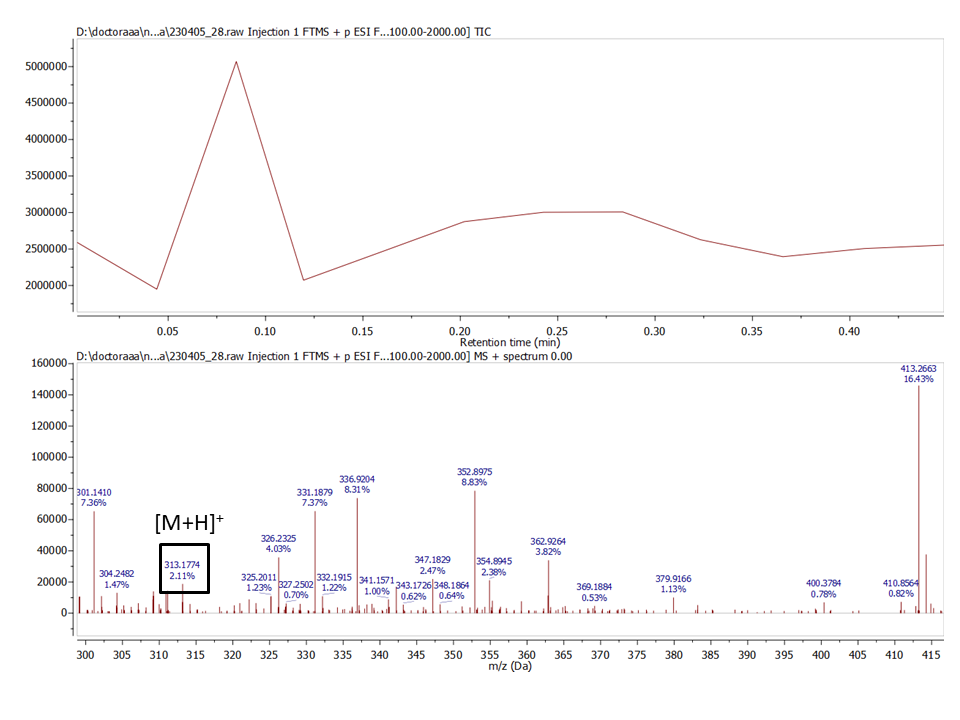
Figure **S23**: ^1^H-NMR spectral data of compound 5 (500 MHz, CDCl_3_).

Figure **S24**: Positive HR-ESI-MS spectrum of compound 5.


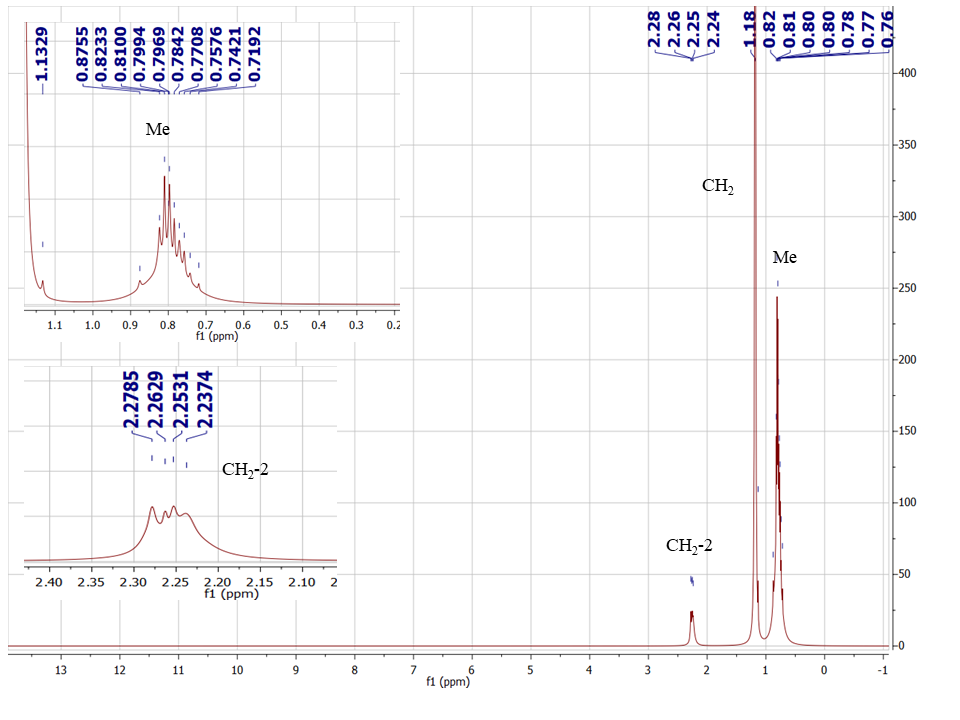


Figure **S25**: ^1^H NMR spectrum of compound **6** (500 MH_Z_, CDCl_3_).


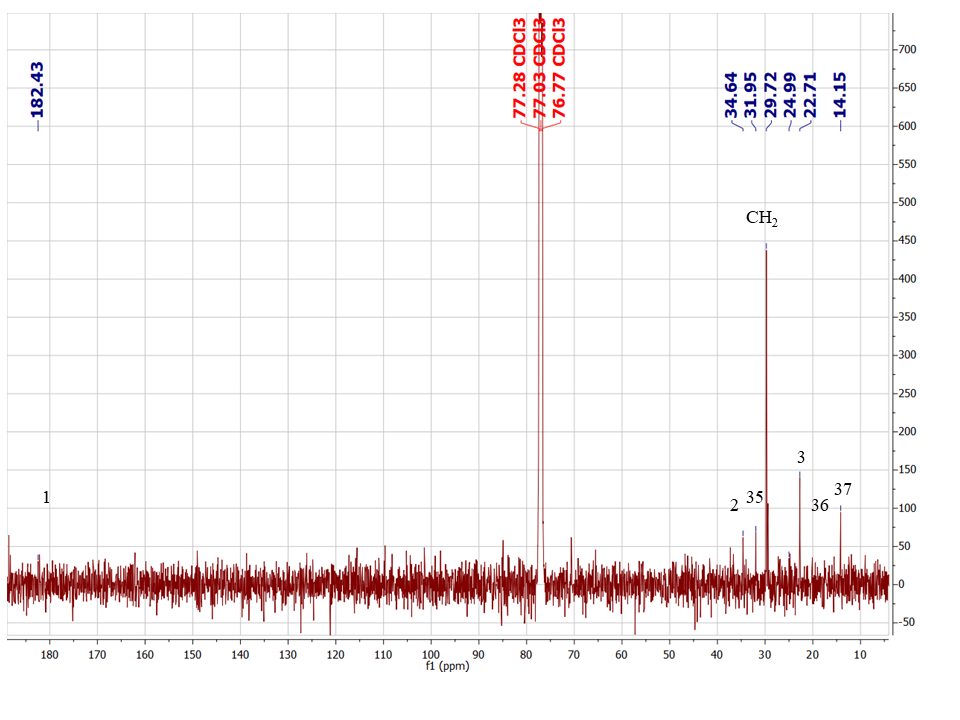


Figure **S26**: ^13^C NMR spectrum of compound **6** (125 MH_Z_, CDCl_3_).


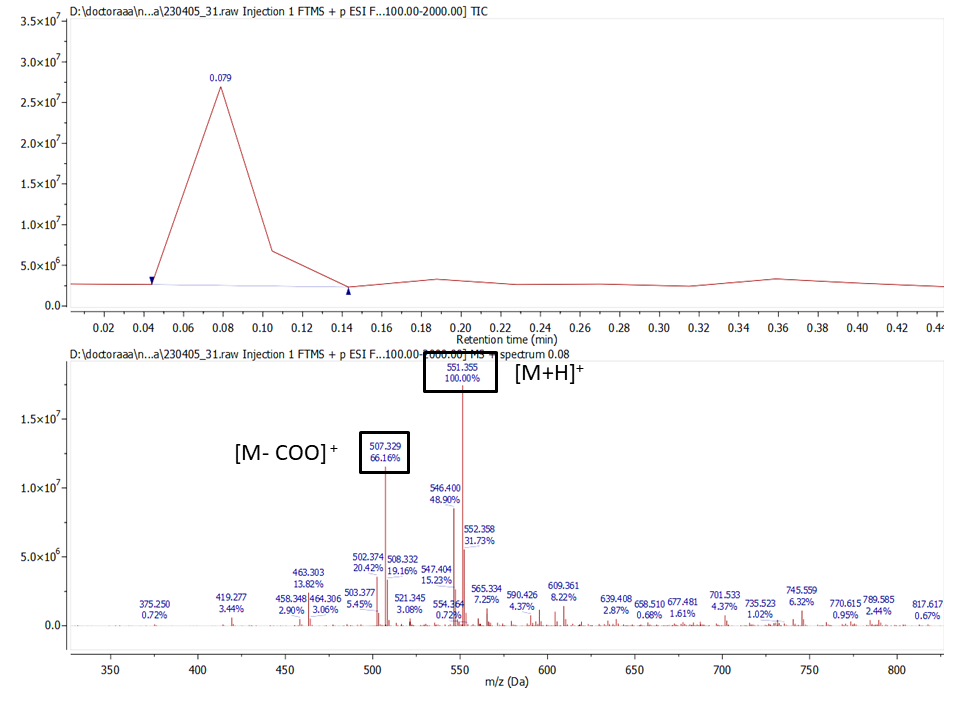


Figure **S27**: Positive HR-ESI-MS spectrum of compound **6**.

Table **S1:** Cytotoxic activity of different fractions against lung cancer cell line (A549)

| **ID** | **Conc.**  ***µ*g/mL** | **O.D** | | | **Mean O.D** | **±SE** | **Viability %** | **Toxicity %** | **IC50**  **± SD** |
| --- | --- | --- | --- | --- | --- | --- | --- | --- | --- |
| A549 | ------- | 0.658 | 0.652 | 0.649 | 0.653 | 0.002646 | 100 | 0 | *µ*g |
| Pet. ether | 1000 | 0.022 | 0.018 | 0.02 | 0.02 | 0.001155 | 3.0627871 | 96.937212 | 169.24 ± 2.62 |
|  | 500 | 0.053 | 0.044 | 0.047 | 0.048 | 0.002646 | 7.3506891 | 92.649310 |  |
|  | 250 | 0.113 | 0.153 | 0.127 | 0.131 | 0.011719 | 20.061255 | 79.938744 |  |
|  | 125 | 0.421 | 0.397 | 0.404 | 0.407333 | 0.007126 | 62.378764 | 37.621235 |  |
|  | 62.5 | 0.625 | 0.638 | 0.611 | 0.624667 | 0.007796 | 95.661051 | 4.3389484 |  |
|  | 31.25 | 0.645 | 0.661 | 0.653 | 0.653 | 0.004619 | 100 | 0 |  |
| Pet.ether- EtOAc (1:1) | 1000 | 0.02 | 0.025 | 0.021 | 0.022 | 0.001528 | 3.3690658 | 96.630934 | 94.55 ± 1.34 |
|  | 500 | 0.018 | 0.022 | 0.019 | 0.019667 | 0.001202 | 3.0117406 | 96.988259 |  |
|  | 250 | 0.046 | 0.041 | 0.058 | 0.048333 | 0.005044 | 7.4017355 | 92.598264 |  |
|  | 125 | 0.197 | 0.203 | 0.173 | 0.191 | 0.009165 | 29.249617 | 70.750382 |  |
|  | 62.5 | 0.465 | 0.451 | 0.449 | 0.455 | 0.005033 | 69.678407 | 30.321592 |  |
|  | 31.25 | 0.642 | 0.635 | 0.65 | 0.642333 | 0.004333 | 98.366513 | 1.6334864 |  |
| EtOAc | 1000 | 0.02 | 0.02 | 0.018 | 0.019333 | 0.000667 | 2.9606942 | 97.039305 | 75.54 ± 0.94 |
|  | 500 | 0.019 | 0.017 | 0.02 | 0.018667 | 0.000882 | 2.8586013 | 97.141398 |  |
|  | 250 | 0.022 | 0.021 | 0.018 | 0.020333 | 0.001202 | 3.1138335 | 96.886166 |  |
|  | 125 | 0.075 | 0.083 | 0.068 | 0.075333 | 0.004333 | 11.536498 | 88.463501 |  |
|  | 62.5 | 0.371 | 0.363 | 0.382 | 0.372 | 0.005508 | 56.967840 | 43.032159 |  |
|  | 31.25 | 0.561 | 0.584 | 0.577 | 0.574 | 0.006807 | 87.901990 | 12.098009 |  |
| EtOAc- MeOH  (1:1) | 1000 | 0.034 | 0.021 | 0.028 | 0.027667 | 0.003756 | 4.2368555 | 95.763144 | 298.66 ± 3.28 |
|  | 500 | 0.055 | 0.063 | 0.062 | 0.06 | 0.002517 | 9.1883614 | 90.811638 |  |
|  | 250 | 0.377 | 0.352 | 0.369 | 0.366 | 0.007371 | 56.049004 | 43.950995 |  |
|  | 125 | 0.587 | 0.574 | 0.587 | 0.582667 | 0.004333 | 89.229198 | 10.770801 |  |
|  | 62.5 | 0.621 | 0.614 | 0.633 | 0.622667 | 0.005548 | 95.354772 | 4.6452271 |  |
|  | 31.25 | 0.65 | 0.661 | 0.647 | 0.652667 | 0.004256 | 99.948953 | 0.0510464 |  |
| MeOH | 1000 | 0.016 | 0.019 | 0.019 | 0.018 | 0.001 | 2.7565084 | 97.243491 | 336.54 ± 6.59 |
|  | 500 | 0.073 | 0.089 | 0.093 | 0.085 | 0.00611 | 13.016845 | 86.983154 |  |
|  | 250 | 0.437 | 0.468 | 0.452 | 0.452333 | 0.00895 | 69.270035 | 30.729964 |  |
|  | 125 | 0.639 | 0.655 | 0.631 | 0.641667 | 0.007055 | 98.264420 | 1.7355793 |  |
|  | 62.5 | 0.642 | 0.649 | 0.654 | 0.648333 | 0.00348 | 99.285349 | 0.7146503 |  |
|  | 31.25 | 0.651 | 0.658 | 0.647 | 0.652 | 0.003215 | 99.846860 | 0.1531393 |  |
